# Cr(I)–Cr(I) Terphenyl Bridged Complexes: A Broken-Symmetry DFT and Multideterminant CASSCF/NEVPT2 Handshake

**DOI:** 10.1021/acsomega.5c06364

**Published:** 2025-10-08

**Authors:** Andrej Hlinčík, Michal Malček, Karol Lušpai, Jozef Kožíšek, Lukas Bucinsky

**Affiliations:** Institute of Physical Chemistry and Chemical Physics, Faculty of Chemical and Food Technology, 61791 Slovak University of Technology in Bratislava, Radlinského 9, SK-812 37 Bratislava, Slovak Republic

## Abstract

In this article, a Cr^I^–Cr^I^-based system stabilized by two monovalent terphenyl ligands Ar′CrCrAr′ (Ar′ = C_6_H_2_-2,6­(C_6_H_3_-2,6-Pr_2_^i^)_2_-4-SiMe_3_) is revisited. It is shown that multideterminant *ab initio* (CASSCF, NEVPT2) and DFT (BLYP, B3LYP as well as ωB97X-D, M06–2X, and B3LYP-GD3) calculations are capable of addressing this system in a consistent way: the spin state ordering, the presence of antiferromagnetic interaction within the Cr^I^–Cr^I^ moiety, and the optimized geometry. Singlet is found to be the ground state by all methods, the broken-symmetry (BS) one in the case of DFT. The singlet CASSCF effective bond order of 3.36 is well resolved by BS singlet BLYP delocalization index (3.15) and Mayer bond order (3.19). The antiferromagnetic interaction is found non-negligible in the CASSCF wave function, with the weight of two open-shell singlet determinants being 31%. In the case of geometry optimizations, singlet NEVPT2 and BS singlet BLYP are able to assess the “experimental” geometry (*d*_Cr–Cr_ ∼ 1.7 Å) while singlet CASSCF and BS singlet B3LYP lead to a longer Cr^I^–Cr^I^ bond length (*d*_Cr–Cr_ > 2.5 Å). The restricted singlet B3LYP and BLYP calculations are energetically disfavored, unstable, and tend to a larger covalency (bond order of four), but address the geometry of the system well, with *d*_Cr–Cr_ values of 1.65 and 1.60 Å, respectively.

## Graphical abstract

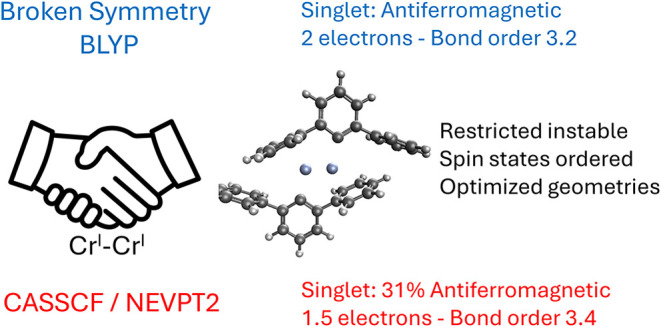

## 1 Introduction

A special place among complexes with a direct metal–metal interaction belongs to the Cr–Cr moiety. The single oxidized Cr^I^ species with an electron configuration of [_18_Ar]­3d^5^ in the presence of two monovalent terphenyl ligands was the first prototype of a complex with the formally quintuple Cr^I^–Cr^I^ bond, see Figure 1. The first ArCrCrAr species (Ar = C_6_H_3_-2,6­(C_6_H_3_-2,6-Pr_2_^i^)_2_, i.e., the monovalent terphenyl ligand), was synthesized by Nguyen et al. in 2005 with a reported Cr^I^–Cr^I^ bond distance of 1.8351 Å.^1^ Except the Cr^I^–Cr^I^ bond, the Cr^I^ cations also interact with the carbon atoms of the terphenyl ligand, i.e., with the deprotonated ipso C atom on the bridging phenyl (the shorter bond, 2.131(1) Å), and with the sp^3^ ipso C atom of the flanking ring (longer bond, 2.2943(9) Å). Wolf et al.^2^ prepared crystals of structures Ar′CrCrAr′ (Ar′ = C_6_H_2_-2,6­(C_6_H_3_-2,6-Pr_2_^i^)_2_-4-X; X = H, SiMe_3_, OMe, and F), which are analogs of the ArCrCrAr complex of Nguyen et al.^1^ All complexes of Wolf et al. have indeed properties similar to those of ArCrCrAr reported in Nguyen et al.; only the Cr^I^–Cr^I^ distance is slightly different, which is not influenced by the substituent, but more likely due to crystal packing effects.^2^ Herein, we choose the silicon-containing complex as the reference template (**4**, X = SiMe_3_) with the Cambridge Crystallography database code SIYNAQ (Figure 1a) and the Cr^I^–Cr^I^ bond length of 1.8077 Å.^2^ These strongly correlated singlet systems are of further interest to validate the performance of computational methods and compare the performance of DFT and *ab initio* approaches, which is of importance for the electronic structure description, spin state energetics preference, and geometry optimization.^3–5^

**Figure 1:**
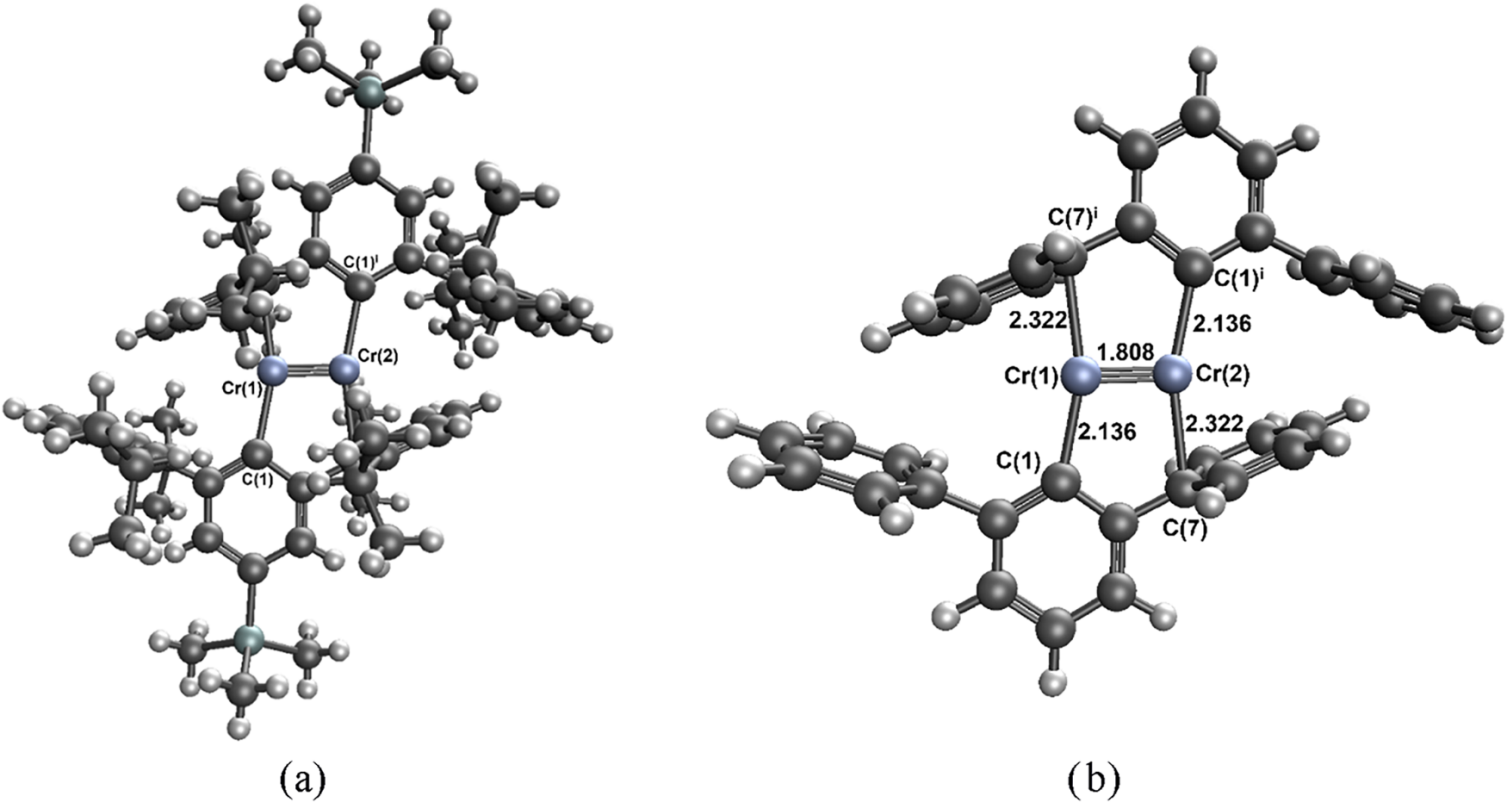
Experimental structure of the SIYNAQ complex (a) and the complex **S** (b).

From a computational point of view, Nguyen et al.^1^ explored the electronic structure of ArCrCrAr by pure GGA (type I) and hybrid DFT (type II) functionals in the restricted regime of the singlet state (closed shell, CS) and mentioned also the potential antiferromagnetic character in this system, as it was suggested for the Cr_2_ dimer as well.^6,7^ The five highest occupied frontier orbitals are of pure Cr^I^–Cr^I^ d-orbital nature, with four molecular orbitals (MO) being in a bonding interaction, while HOMO shows almost no overlap for the chosen isosurface, suggesting a physical bond order lower than 5 even in the restricted regime. The extended scheme with natural orbitals for the chemical valence density decomposition approach (ETS-NOCV method) concluded a quintuple Cr^I^–Cr^I^ bond for the restricted regime DFT of Ar^S^CrCrAr^S^ (Ar^S^ = C_6_H_3_-2,6­(C_6_H_5_)) and ArCrCrAr.^8^ The pure GGA DFT geometry optimizations in the restricted singlet state regime did successfully resemble the crystal structure for Ar′CrCrAr′ complexes of Wolf et al. (the optimized *d*_Cr–Cr_ bond length was shorter by about 0.1 Å).^2^ Brynda et al.^9^ also studied a model structure of the PhCrCrPh system (Ph = phenyl) in two configurations, the linear and trans-bent one, including a comparison to the Cr_2_ dimer. The reported B3LYP DFT calculations in RKS and UKS regimes (CASPT2 optimized geometry) yield larger bond orders in the restricted closed-shell regime (around 4 for the complexes studied and around 5 for the Cr_2_ dimer itself) compared to the broken-symmetry (BS) singlet state unrestricted case with lower bond orders (lower than 3),^10^ see Table 1. It should be noted that the authors acknowledge the problematic description of strongly correlated systems by hybrid DFT functionals.^10^ Nevertheless, the authors were able to correct the obtained potential energy curve of the BS-DFT calculation of the Cr_2_ dimer by the use of the Noodleman^11,12^ and Yamaguchi^13,14^ schemes.^10^ Importantly, the singlet spin state is confirmed as energetically preferred in the reported CASSCF/CASPT2 calculations. From geometry optimizations at the Douglas-Kroll-Hess (DKH) CASSCF/CASPT2­(14,14) level of theory, the authors report an effective bond order (EBO) value of 3.52 for the trans-bent structure with Cr^I^–Cr^I^ bond distance of 1.752 Å. Albeit it was still argued that it is formally a quintuple bond, i.e., all Cr d-orbitals are involved, but the occupation of the antibonding orbitals lowers the EBO.^10^ The main configuration with a 45% weight in the CASSCF wave function was the one with all 3d bonding orbitals occupied. The second largest contribution, with a 9% weight, belonged to a double excitation from 3δ orbitals to their antibonding counterparts.

**Table 1:** Bond Distances (*d*_Cr–Cr_), EBO, and WBO Values from Previous Studies

method	system	*d*_Cr–Cr_/Å	EBO	WBO^i^
CASPT2(14,14)/DKH^a^	Cr_2_	1.679 ^10^	4.45 ^10^	6.03^CS^ ^10^, 3.46^BS^ ^10^
	MeCrCrMe	1.849 ^15^	2.96 ^15^	4.94 ^15^
	PhCrCrPh^c^	1.752 ^9^	3.52 ^9^	4.77 ^15^
	PhCrCrPh^d^	1.678 ^9^	3.69 ^9^	4.82^CS^ ^10^, 3.79^BS^ ^10^
CASPT2(14,14)/DKH^b^	ArCrCrAr^e^	1.835 ^1^	3.43 ^15^	4.12^CS^ ^10^
BLYP/TZP/ZORA^a^	Ar^§^CrCrAr^§^ ^f^	1.736 ^15^		
	Ar^#^CrCrAr^#^ ^g^	1.744 ^15^		
BVP86/TZP/ZORA^a^	ArCrCrAr^e^	1.725 ^2^		
	Ar*CrCrAr*^h^	1.721 ^2^		

a Geometry optimization.

b Single-point calculation of X-ray geometry.

c Trans-bent.

d Linear.

e Ar = C_6_H_3_-2,6­(C_6_H_3_-2,6-Pr_2_^i^)_2_.

f Ar^§^ = C_6_H_4_-2­(C_6_H_5_).

g Ar^#^ = C_6_H_3_-2,6­(C_6_H_3_-2,6-Me_2_)_2_.

h Ar* = C_6_H_1_-2,6-(C_6_H-2,4,6-^i^Pr_3_)_2_.

i B3LYP calculation.

The EBO of the Cr^I^–Cr^I^ bond was later calculated to be 3.43 by DKH CASSCF­(14,14) calculations for the ArCrCrAr system.^15^ The Cr_2_ dimer itself (Cr atom electron configuration of [_18_Ar]­4s^1^3d^5^) and a formal sextuple bond (σ^4^π^4^δ^4^) has a short Cr–Cr distance 1.6788 Å,^16^ a low dissociation energy (35.3 ± 1.4 kcal mol^–1^),^16^ and a CASSCF EBO of 4.51.^10^ Such EBO value of the Cr–Cr bond in the Cr_2_ dimer closely matches the occupancy of the antibonding natural orbitals at the equilibrium geometry.^9,10^ For comparison, the formal bond in Cu_2_ dimer has a larger dissociation energy (47.93 ± 0.57 kcal mol^–1^) and a longer bond distance (2.2193 Å).^17–19^ For completeness, Cr^I^–Cr^I^ bond distances, EBO, and Wiberg bond order (WBO) values from previous studies are compiled in Table 1.

It is necessary to mention that there are various options for the chemical variation of ligands stabilizing the Cr^I^–Cr^I^ bond.^20^ These ligands are mostly based on diazadiene,^21^ amidinate,^22^ and guanidinato^23^ type of ligands, leading to variation in the Cr^I^–Cr^I^ bond distances and EBO.^24^ Additional model ligand suggestions for even shorter Cr^I^–Cr^I^ bond distances were based on DFT calculations.^25^ The potential energy curves presented by Huang et al.^26^ for a symmetric diazadiene type of such complex (Ar^N^CrCrAr^N^) are considered an admixture of covalent (EBO 3.73) and antiferromagnetic interactions, with their weights depending on the actual Cr^I^–Cr^I^ distance.^27^ This is in fact true for Cr_2_ dimer as well,^28^ a covalent bond is manifested at short distances and the antiferromagnetic coupling at larger separations. Very importantly, it is mentioned that the restricted singlet spin state DFT calculation has an internal instability (hybrid functionals, B3LYP) except in the cases where the overlap of the restricted and BS curves is found in the optimized geometries of diazadiene type of Cr^I^–Cr^I^ complexes (pure GGA functionals, BP86).^26^ It is further suggested to use pure GGA functionals (type I) in the restricted singlet state regime to assess the optimized geometries and hybrid (or meta GGA) functionals (type II) in the BS singlet state regime to analyze the bonding properties and the electronic structure.^26^ In addition, a similar double potential well shape is found for Cr^II^–Cr^II^ of the dichromium tetrakis (μ-acetato)-diaqua complex with a CAS EBO of 0.8 and UBLYP BS singlet delocalization index (DI) of 1.054 ^29^ and an experimental *d*_Cr–Cr_ of 2.348 Å.^30^ The type I and type II DFT functionals show to prefer short (covalent) or long (antiferromagnetic) type of interaction even in this Cr^II^–Cr^II^ system,^29^ respectively. Farrugia and Macchi^31^ reviewed the correlation between formal bond order in complexes with M–M interactions and the parameters from the topological analysis of quantum theory of atoms in molecules (QTAIM).^32^ It was clearly shown that the closest parameter to compare with the formal bond order is delocalization index (DI), including the Cr^I^–Cr^I^ and Cr^II^–Cr^II^ systems mentioned above. In the case of multiple M–M bonds, DI is always smaller than the formal bond order.

In the presented work, we will point out that there are much more common features for the BS-DFT singlet and CAS singlet electronic structure than one would expect, even though the use of a one-determinant approach of the DFT ansatz is known to be problematic for the description of strongly correlated systems.^5,33,34^ The SIYNAQ complex is considered as the reference template (Ar′ = C_6_H_2_-2,6­(C_6_H_3_-2,6-Pr_2_^i^)_2_-4-SiMe_3_), see Figure 1a, with the major focus on a smaller system (Ar^S^ = C_6_H_3_-2,6­(C_6_H_5_)), denoted as **S**, see Figure 1b. Energetics of the rigid experiment-like geometry are evaluated for pure GGA (type I, BLYP) and hybrid DFT (type II, B3LYP) functionals and compared to advanced DFT congeners (ωB97X-D, M06-2X, and B3LYP-GD3). The comparison of restricted closed shell and BS unrestricted singlet calculations is reported. All relevant spin states are considered for completeness, including their stability consideration. The size of the system (SIYNAQ vs **S**), relativistic effects (DKH2 vs NR), and basis set quality (def2-SVP vs def2-TZVPP), as well as the DFT calculation stability are considered. Besides energetics, the Cr^I^–Cr^I^ bond is characterized by means of QTAIM analysis (focusing on the DI, a QTAIM bond order equivalent, including charges/spins comparison with respect to Mulliken populations), and Mayer bond order (MBO) to compare with the literature. In addition, CASSCF/NEVPT2 energetics of the different spin states are evaluated, EBO is considered, and the diamagnetic and antiferromagnetic contributions in the CASSCF wave function configurations are analyzed to correlate these findings with DFT results. Geometry optimizations of all relevant spin states at the DFT level are considered and compared to CASSCF geometry optimizations, as well as to a CASSCF potential energy scan of the Cr^I^–Cr^I^ distance with NEVPT2 energies being evaluated.

## 2 Computational Details

To avoid the computational overhead when treating the whole SIYNAQ complex (see Figure 1a), the tetraisopropyl and trimethylsilyl groups were replaced with hydrogen atoms to obtain the model complex denoted as **S**, i.e., Ar^S^CrCrAr^S^ (Ar^S^ = C_6_H_3_-2,6­(C_6_H_5_)), see Figure 1b. The C–H bond lengths were set at 0.925 Å first. Then, the relaxation of hydrogens was done in the Gaussian16 package^35^ with the B3LYP functional^36–39^ and def2-SVP basis set^40^ (for the energetically preferred BS singlet spin state, see below). In addition, range-separated ωB97X-D,^41,42^ meta-hybrid M06-2X,^43^ and dispersion-corrected B3LYP-GD3 ^44^ functionals have been accounted for to test other functional types and extensions. If not otherwise stated, all of the presented results are relevant to the **S** complex. Since chromium is formally in the oxidation state one (I), corresponding to a d^5^ electronic configuration, all spin states up to S = 5 were considered.

Subsequently, DFT *in vacuo* single-point calculations were performed using BLYP^36,37,45^ and B3LYP functionals and the def2-SVP basis set. In the case of unrestricted calculations, UBLYP and UB3LYP were utilized, which allow assessment of the BS regime for the unrestricted singlet spin state. The def2-TZVPP basis set^40^ was also used to evaluate the energy differences and to confirm the obtained def2-SVP basis set results. These calculations were performed in the Gaussian16 (all spin states) as well as ORCA 4.2.0 and 5.0.3 ^46–48^ program packages (only low- and high-spin states) to cross-check the results. The resolution of identity approximation (RI)^49^ was used with the def2/J auxiliary basis set^50^ and the RIJCOSX algorithm^51^ for the B3LYP functional in ORCA. The spin state preference was evaluated in a way, where all spin states were cross-checked with being restarted from any other spin state in the mix, including a stability inspection of each considered DFT calculation against the “stable” keyword as implemented in Gaussian16.^52^ Because of the multiconfigurational character of the Cr^I^–Cr^I^ interaction in the studied complex, the state-specific CASSCF^53–55^ (from hereafter abbreviated as CAS) method was used as available in ORCA. State-averaged CAS calculations have been performed to exclude the presence of near-degeneracy issues. The CAS space was built from Cr d-like orbitals (using UBLYP localized orbitals for the high or BS singlet spin states as input active space orbitals), with 10 electrons correlated in 10 orbitals, designated as CAS­(10,10). To evaluate also the dynamic correlation on top of the CAS­(10,10) calculation, the NEVPT2 method was used.^56–59^ Additionally, CAS­(10,15) calculations were performed to consider the impact of a larger active space on the Cr^I^–Cr^I^ interaction using the ExtOrbs DoubleShell keyword. The impact of DKH second order (DKH2) scalar relativistic calculations as implemented in the Gaussian16 and ORCA program packages^60–64^ was evaluated for the jorge-DZP-DKH basis set.^65–67^ DKH2 BLYP and B3LYP calculations in ORCA program were using the RI approximation with the SARC/J auxiliary basis set.^68,69^ Geometry optimizations of all spin states were performed at the BLYP and B3LYP level of theory (Gaussian16) as well as for the CAS­(10,10) level of theory using the def2-SVP basis set with RI and auxiliary basis set def2/JK^70^ in the ORCA program. In addition, a potential energy surface scan was performed for the Cr^I^–Cr^I^ distance at the CAS­(10,10) level of theory. Herein, the Cr^I^–Cr^I^ distance was scanned from 1.4 to 3.6 Å (step size length has varied, see the Geometry Optimizations section), with relaxing the rest of the geometry of **S**, including a NEVPT2 calculation for the relaxed geometry at each frozen Cr^I^–Cr^I^ distance, in both cases with the RI approximation accounted for.

To characterize the Cr^I^–Cr^I^ interaction further, Bader’s QTAIM analysis was used.^32^ The input files for these calculations were fchk files from Gaussian16, and gbw to (orca_2mkl) molden to (molden2aim^71^) wfn transformed ORCA files. QTAIM analysis was performed with the AIMAll program package.^72^ Orca_plot program was used for the creation of cube files of CAS­(10,10) natural orbitals and spin densities which were visualized in the IQmol program.^73^

## 3 Results and Discussion

### 3.1 Single-Point Calculations—Energetics

Energetics of different spin states from DFT (UBLYP and UB3LYP) and multiconfigurational CAS/NEVPT2 method for complex **S**, based on the experimental geometry, is shown in Table 2 (nonrelativistic Hamiltonian and the def2-SVP basis set). Total energies are compiled in Table S1 for completeness. When comparing DFT and CAS/NEVPT2 results, it must be emphasized that all of the methods yield the same energy ordering for the different spin states. The energy difference between the S = 0 (low-spin) and S = 5 (high-spin) states of UBLYP calculation correlates well with NEVPT2 calculations. It is to be noted that the multiconfigurational treatment has a different reference space (number of configurations) available for the S = 5 calculation (one configuration/determinant) and for the S = 0 case (8953 configurations or 19404 configuration state functions/determinants). This is why state-specific CAS calculations with S = 5 and 0 were obtained to see the impact of the reference space on the relative energies of different spin states. The state-averaged calculations did not lead to any degeneracy issues, i.e., the ground state singlet is well resolved, and the second singlet state is above the first four (or five) triplet and the lowest quartet states, see Table S2. The CAS­(10,10) and NEVPT2 energetics of state-specific and state-averaged calculations for 5 lowest roots of each spin state (except S = 5 with only one root) are shown in Table S2.

**Table 2:** Relative Energy of Different Spin States Δ*E* (in kJ mol^–1^) of Complex **S** for the Used Methods: UBLYP, UB3LYP, CAS­(10,10), and NEVPT2 with the def2-SVP Basis Set

			S = 5		S = 0		Huang et al.^26^
S	UB3LYP	UBLYP	CAS(10, 10)	NEVPT2	CAS(10, 10)	NEVPT2	RASPT2-E
0	0.00	0.00	0.00	0.00	0.00	0.00	0.00 ^26^
	(190.97)^a^	(26.82)^a^					
1	103.94	66.87	41.71	51.90	67.34	65.87	105.44 ^26^
2	154.64	118.34	116.58	143.65	183.29	171.22	230.54 ^26^
3	299.25	335.69	231.54	308.18	372.66	363.95	436.39 ^26^
4	465.88	576.93	355.16	490.29	572.87	575.91	665.26 ^26^
5	626.28	805.05	479.20	678.23	770.12	792.08	910.44 ^26^

a Restricted calculation; the last column compiles data from Huang et al. diamidinate Cr^I^–Cr^I^ complex^26^ at the equilibrium geometry

The spin state energetics (spin preference) of complex **S** confirms the singlet as the ground state for both CAS/NEVPT2 and DFT calculations, in full agreement with the previous studies.^6,9,10^ In the case of DFT, it is the BS singlet spin state as was also reported in the previous papers.^1,10^ The BLYP (B3LYP) restricted vs BS unrestricted energy difference is ∼30 (190) kJ mol^–1^. The restricted singlet spin state is found to be unstable, as previously presented in Huang et al. for a similar diazadiene complex.^26^ It is also found that DFT results of symmetric spin configurations with respect to Cr atoms for all spin states lower than S = 5 are found to be unstable. All stable DFT intermediate spin states (except S = 2) have an uneven BS spin distribution on the Cr atoms, see Tables S3 and S4. Still, the unstable Cr^I^–Cr^I^ intermediate symmetric spin states are close in energy (within 3 kJ mol^–1^) to the BS cases for S = 1 and S = 3 (BLYP), and S = 3 and S = 4 (B3LYP), see Tables S3 and S4. The unicity of DFT B3LYP solutions has been cross-validated via restarting each spin state from all the other spin states using both guess = read and guess = (read, mix) keywords; for BLYP, only a few cases have been validated. The ground state for each spin state is found to be consistent and robust, and no intermediate states have been found.

Relativistic DKH2 calculations of **S** do not affect the order of the spin states, just increase the energy differences between the low- and high-spin states by up to 20 (50) kJ mol^–1^ at the DFT (*ab initio*) level of theory, see Tables S5, S6, and S7. In a similar fashion, the size adjustment of the initial full scale SIYNAQ complex to the model complex **S** was verified at the DFT level of theory (BLYP and B3LYP, def2-SVP), see Tables S8 and S9, as well as the performance of the def2-SVP basis set in comparison to the def2-TZVPP basis set (BLYP and B3LYP, **S** complex), see Tables S10 and S11. The energy difference between the high- and low-spin states is almost the same for the SIYNAQ complex compared to the **S** complex calculations (i.e., less than 10 kJ mol^–1^). The use of the def2-TZVPP basis set compared to def2-SVP leads to a 30 kJ mol^–1^ lower energy difference of the high vs low DFT spin states. It is also worth noting that the Mulliken charges and spins on Cr ions do not change significantly when considering the impact of system size, relativistic effects, or the basis set quality. This is interesting due to the known dependence of Mulliken population results, especially upon the basis set quality. When considering the energetics for the additional functionals used (ωB97X-D, M06-2X, and B3LYP-GD3), the picture remains essentially very close to that of B3LYP; see Tables S12–S14. B3LYP-GD3 is almost the same as B3LYP (no intermolecular interaction at all), and the other (meta) hybrid functionals do not change the significance of the results either.

### 3.2 Single-Point Calculations—Electronic Structure

The DFT and CAS descriptions of the singlet spin state in the realm of electronic structure will be considered for the Cr^I^–Cr^I^ bond character/order. In the case of CAS, it is the effective bond order (EBO), and in the case of DFT, the QTAIM delocalization index (DI) and Mayer bond order (MBO) are used. The herein calculated CAS­(10,10) EBO is 3.363 (complex **S**), which suggests a triple (less than 3.5) bond character as found previously in this kind of complexes studied at the CASSCF level of theory, see Table 1. The EBO is defined as^74^

1 $$EBO=\frac{\sum \left(n_{bonding}-n_{antibonding}\right)}{2}$$

where *n*_bonding_ represents occupation numbers of bonding natural orbitals and *n*_antibonding_ represents occupation numbers of antibonding natural orbitals of the 10 d-like CAS orbitals. Natural orbitals and occupation numbers of the CAS­(10,10) calculation are listed in Figure 2. UBLYP BS S = 0 state has DI equal to 3.146, which confirms a triple bond character (UB3LYP BS S = 0 leads to DI equal to 2.269), see Table 3. MBOs correlate with the obtained DI results, suggesting a triple bond for BS S = 0 state in the UBLYP calculation, while a double bond is found in the UB3LYP calculation; see Table 3.

**Figure 2:**
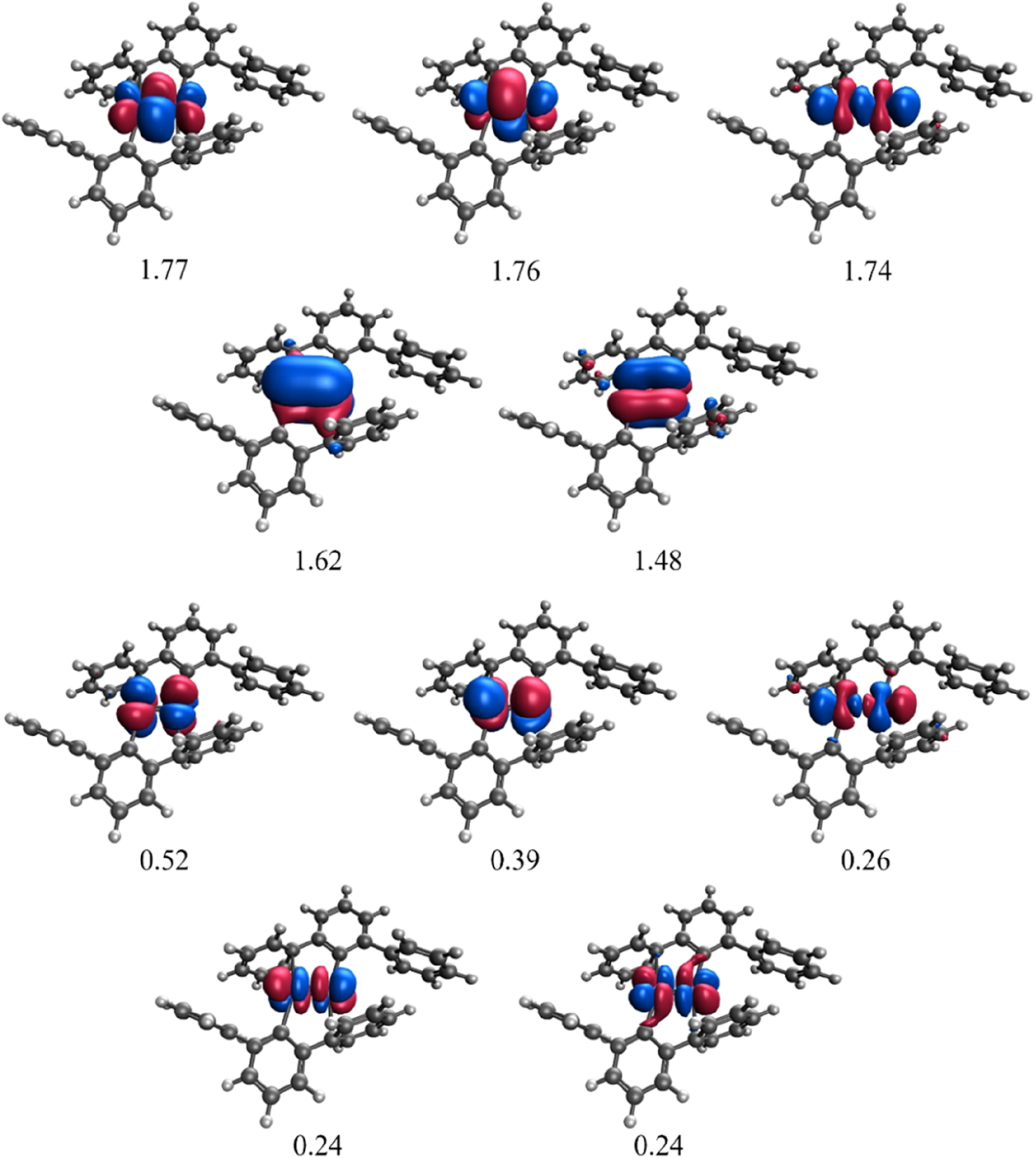
CAS­(10,10)/def2-SVP S = 0 natural orbitals and occupation number of complex **S**.

**Table 3:** QTAIM Atomic Charge and Spin Populations at Cr Atoms, DI, MBO Values for Chosen DFT Calculations, and CAS­(10,10) MBO and EBO Values of Cr^I^–Cr^I^ Bond

method	spin state	QTAIM charge	QTAIM spin	DI	MBO	EBO
UB3LYP	S = 0	0.657	3.127	2.269	2.159	
	S = 5	0.724	4.587	3.980	4.149	
	S = 0 (R)	0.645		0.650	0.378	
UBLYP	S = 0	0.642	1.983	3.146	3.194	
	S = 5	0.734	4.417	3.950	4.134	
	S = 0 (R)	0.636		0.720	0.524	
CAS(10,10)	S = 0	0.709		18.012^a^	2.278	3.363
	S = 5	0.736	4.721	0.530	0.1<	

a Failure due to the approximated treatment of two-electron density matrix contributions of the multideterminant wave function.^80,81^

Aside from the CAS­(10,10) EBO value, a closer examination of CAS­(10,10) determinants (S = 0 calculation) of the singlet spin state shows a 64% closed-shell character (abbreviated as CSC, i.e., the sum of the weights of determinants with only doubly occupied or empty orbitals). The highest 45% weight in the CAS­(10,10) wave function has the determinant [2222200000] in agreement with previously published results.^10^ The CAS wave function is further composed of 31% of singlet determinants that have two electrons excited (leading to four unpaired electrons, e.g., [2221111000]), and of 3% of singlet determinants with four electrons excited (leading to eight unpaired electrons). The total sum of CAS­(10,10) wave function determinants evaluated was 98% for the threshold of 5 × 10^–4^, and the five main configurations of each spin state can be found in Table S15. Importantly, all the two- and four-electron excited singlet determinants lead to configurations, fully canceling the spins on the Cr atoms and yielding a pure closed-shell singlet CAS­(10,10) wave function. Nevertheless, these results suggest that the open-shell character cannot be neglected in the CAS­(10,10) wave function (31% out of 5 electrons leads to a value of 1.5 on each chromium). The composition of the CAS determinants at the DKH level of theory is almost the same (with a 2% smaller open-shell character found). QTAIM Cr^I^ spin populations for the BS UBLYP calculation are 1.98 and −1.98. Thus, three electrons on each Cr^I^ cation contribute to the closed-shell interactions for UBLYP. The BS UB3LYP calculation yields larger Cr^I^ spin populations (3.13 and −3.13) reflecting the smaller bond order, see Table 3. Note that the expansion of CAS singlet (S = 0) in the high-spin S = 5 solution yields a wave function that is composed of 43% high spin, 31% a single double shell, and 13% of two double shell configurations (MBO = 1.70 and energy destabilization with respect to the CAS­(10,10) state-specific singlet of 99 kJ/mol). The larger Cr^I^ spin population for UB3LYP BS singlet leads to a larger spin contamination of 2.7 compared to the 1.4 UBLYP value, see Tables S2 and S4. The larger CAS­(10,15) calculation yields an EBO value of 3.54, with the additional five d-orbitals of Cr^I^–Cr^I^ bonding character, see Figure S1. The CAS­(10,15) two-electron excitations (e.g., [222111100000000]) have a 28% contribution.

In general, QTAIM Cr charges (around 0.650) are found closer to the formal charge of Cr^I^ as opposed to the smaller Mulliken charges (around 0.150). QTAIM Cr spins are by 0.3 smaller than Mulliken spins, but in total, Mulliken spins of the Cr atoms are not as sensitive to the basis set and are similar to QTAIM ones. Other bond-related QTAIM characteristics, such as the bond critical point (BCP) electron densities ρ_BCP_, Laplacians *∇* ^2^ρ_BCP_, and ellipticities *ε* for all spin states, can be found in Tables S16–S19 and Figures S2–S4. Interestingly, Cr(1)–C(7)^i^ BCP ellipticity (*ε*) is quite large (0.5), possibly due to stress on the (Cr(2)–Cr(1)–C(7)^i^–C­(2)^i^–C­(1)^i^) ring or the weak bond character between Cr^I^–C. Use of the larger def2-TZVPP basis set (only BS singlet is provided) does not affect the QTAIM parameters significantly, charge and spin change by less than 0.1, see Tables S16–S19. The BS UBLYP spin density is shown in Figure S5.

For the restricted S = 0 closed-shell DFT calculations, DIs are close to 4.0 (both BLYP and B3LYP).^1^ Ponec et al.^27^ also conclude about the presence of a quadruple bond in the restricted DFT regime, with a nonbonding contribution of the fifth electron pair for an alternative complex with a nitrogen coordinating ligand using the Domain average Fermi hole approach. The DI values for all other spin states and Cr^I^–Cr^I^ and Cr^I^–C interactions are compiled in Table S17. Last but not least, DI for the CAS S = 0 wave function is a failure (with the value of 18), which can be attributed to the mismatched two-electron density matrix contribution and the multideterminant character of the system.^75–78^ MBO calculated for the CAS­(10,10) method is only 2.278, suggesting an underestimated value when compared to EBO, which can be also assigned to the lacking of the two-electron density matrix contributions evaluation.^79^ The other tested functionals (ωB97X-D, M06-2X, and B3LYP-GD3) did not lead to any difference in the electronic structure (Mulliken charges) compared to B3LYP, with B3LYP-GD3 being actually the same, see Tables S12–S14.

### 3.3 Geometry Optimizations

The *in vacuo* geometry optimizations of the **S** complex were studied by the same methodologies as in the single-point calculations, DFT (BLYP and B3LYP) and CAS­(10,10)/NEVPT2 with the def2-SVP basis set. Optimized bond distances between Cr(1)–Cr(2) ions, Cr(1)–C(1), and Cr(1)–C(7)^i^ are shown in Tables 4, S20, and S21. Selected dihedral angles are shown in Table S22, and optimized geometries are shown in Figures S6–S8. The use of B3LYP functional elongates the Cr^I^–Cr^I^ distance by 0.7 Å while the BLYP functional shortens this bond by 0.1 Å, when compared to the experimental value, for the S = 0 BS spin state as expected,^10,26,29^ i.e., pure GGA (type I) functionals prefer the covalent (short) interaction region while hybrid (type II) functionals prefer the antiferromagnetic (long) interaction region. Even in the DFT geometry optimization calculations, the BS singlet spin state is confirmed as the ground state, e.g., restricted singlet is ca. 10 (230) kJ mol^–1^ and triplet ca. 50 (50) kJ mol^–1^ above the BS BLYP (B3LYP) singlet, see Tables S23 and S24. The ωB97X-D, M06-2X, and B3LYP-GD3 functionals did not lead to any significant difference in the optimized structures compared to B3LYP, with B3LYP-GD3 being the very same; see Tables S25 and S26. The restricted closed-shell singlet spin state geometry optimization leads in all DFT methods to the shortest Cr^I^–Cr^I^ bond lengths, yet an unstable state. Mulliken spins correlate with the distance and point on the considerable Cr^I^–Cr^I^ bond weakening in the BS singlet spin state of the B3LYP functional optimization showcasing an antiferromagnetic interaction, see spin populations in Table 4. On the other hand, the BLYP calculation amplifies the bond strength, with the bond becoming 0.1 Å shorter. In all DFT methods, geometry optimization for the S = 5 spin state causes breaking of the Cr^I^–Cr^I^ bond. The S = 1 spin state of BLYP geometry optimizations also leads to a covalent interaction geometry, although being energetically disfavored; see Tables S23 and S24.

**Table 4:** Optimized Distances between Chosen Atoms and Mulliken Spins of Cr^I^ Cations for States S = 0 (BS) and S = 5 for Different Methods (min Represents Minima)

		*d*/Å				
method	M	Cr(1)–Cr(2)	Cr(1)–C(1)	Cr(1)–C(7)^i^	spin(Cr(1))	spin(Cr(2))
**S**		1.807	2.136	2.322		
UB3LYP/def2-SVP	1	2.585	2.117	2.574	4.699	–4.699
	11	4.145	2.128	2.991	4.937	4.943
	1(R)	1.601	2.115	2.775		
UBLYP/def2-SVP	1	1.718	2.113	2.426	1.887	–1.893
	11	2.943	2.121	2.492	4.626	4.830
	1(R)	1.655	2.115	2.378		
CAS(10,10) (min)^a^		3.000	2.303	3.334		
NEVPT2 (min)^a^		1.700	2.241	2.772		

a Geometries from PES scan.

CAS­(10,10) geometry optimization (either low spin or high spin) causes the same bond elongation as the B3LYP calculation when not taking into account the dynamic electron correlation, as shown in the literature.^10^ For this reason, an additional CAS­(10,10) potential energy scan for the Cr^I^–Cr^I^ distance was performed (see Figure 3), including a NEVPT2 energy evaluation of each relaxed geometry. Table 4 summarizes the bond lengths in the minima of the potential energy surface scan of the Cr^I^–Cr^I^ distance, and Figure 3 shows the actual CAS­(10,10) and NEVPT2 curves. NEVPT2 calculation is close to the “experimental” geometry of **S** and correlates with the UBLYP BS result. For NEVPT2 geometry with minimum energy, the MBO of the Cr^I^–Cr^I^ bond was found to be 2.781, and EBO has a value of 3.659, with the NEVPT2 minimum being slightly shorter than the BS singlet UBLYP result, see Table 4. Five main configurations of the CAS­(10,10) S = 0 calculation at a *d*_Cr–Cr_ distance of 2.5 Å are shown in Table S27. The composition of natural orbitals for complex **S** for the experimental geometry, the NEVPT2 minimum (*d*_Cr–Cr_ distance of 1.7 Å), and the relaxed geometry (*d*_Cr–Cr_ distance of 2.5 Å) are presented in Table S28.

**Figure 3:**
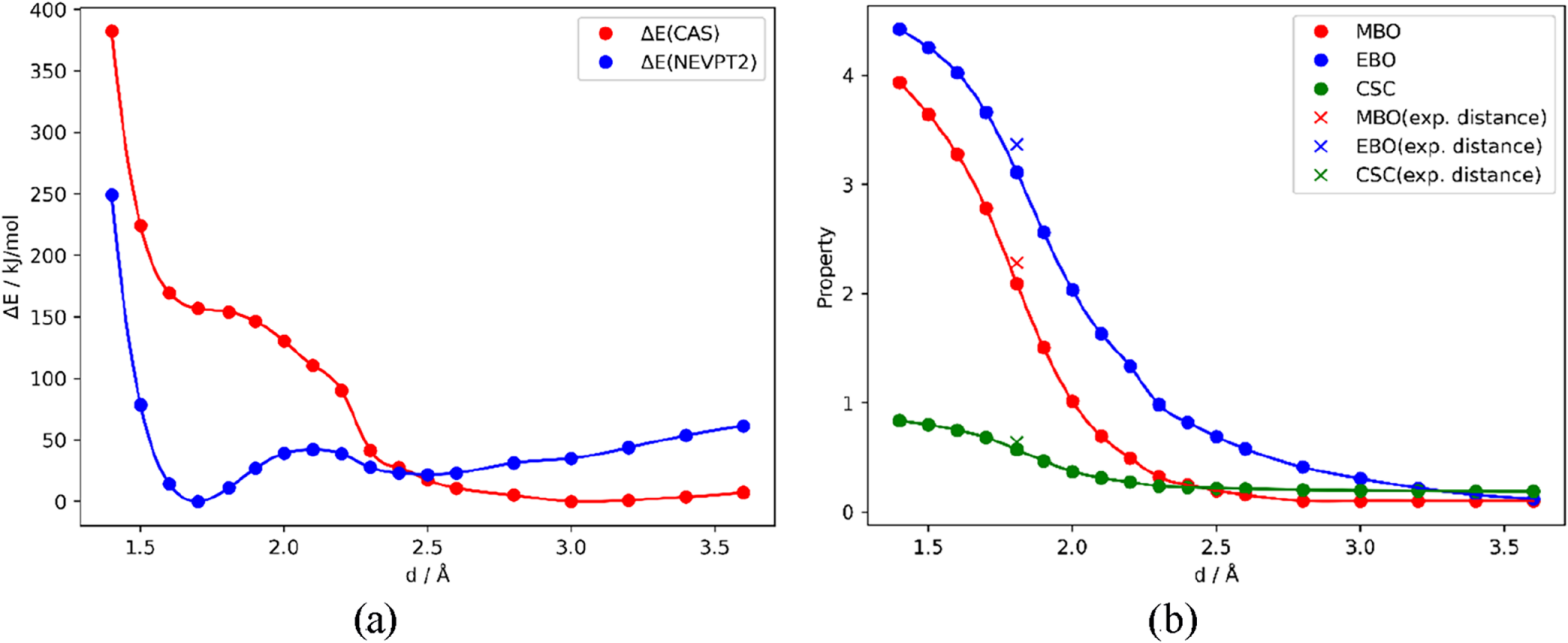
(a) Potential energy curves for CAS­(10,10) and NEVPT2 calculation with def2-SVP basis set for complex **S** and (b) dependence of EBO (Effective bond order), MBO (Mayer bond order), and CSC (Closed-shell character) on the Cr^I^–Cr^I^ distance from CAS­(10,10) S = 0 scan.

The NEVPT2 potential energy curve (PEC) scan is similar to the PEC presented by Huang et al.,^26^ where the inner well is considered to represent covalent bonding while the shoulder represents an antiferromagnetic interaction, see Figure 3a. PECs of the other spin states are presented in Figure S9, including the scans around the minima of DFT methods for all spin states. The dependence of bonding parameters (MBO, EBO, and CSC) on the Cr^I^–Cr^I^ distance for the CAS/NEVPT2 PEC is shown in Figure 3b. All three parameters decrease with the increasing Cr^I^–Cr^I^ bond length in a very similar “S-bend shaped” fashion. This clearly agrees with the large covalent interaction for short Cr^I^–Cr^I^ distances and the antiferromagnetic interaction at larger distances. At this point, the usage of the def2-SVP basis set and the size of the active space at CASSCF calculation needs to be emphasized. Both factors have an impact on the PEC shape, e.g., the formation of a second well at 2.5 Å. To obtain an improved PEC shape, the inclusion of 3d double shell (CAS­(10,20)) and the usage of a larger basis set are recommended.^82^ Despite this, the region of the global minimum is well addressed for the smaller basis set and active space, which can be seen in Figure S10, where the PEC of the diamidinate Cr^I^–Cr^I^ system of Huang et al. is presented^26^ with the basis set enlarged (def2-TZVPP).

## 4 Conclusions

Singlet (BS for DFT) was found to be the ground state by all of the methods used: DFT, CAS­(10,10), and NEVPT2. All methods suggest the same spin state energy ordering, from the low-spin state to high-spin state configuration, and the energy differences are in an acceptable agreement. The formally quintuple Cr^I^–Cr^I^ bond in **S** (SIYNAQ) can be characterized as a triple bond with antiferromagnetic coupling of nonbonded electrons (CAS EBO is 3.36, and BS BLYP leads to DI and MBO of 3.15 and 3.19, respectively). In addition, the BS BLYP antiparallel spin population of two on the Cr^I^ centers matches the CAS 31% antiferromagnetic interaction between the Cr centers. The EBO value corresponds well with the previously published data.^9,10,15^ The restricted singlet DFT results lead to an unstable state of a bond order (DI, MBO) close to four as found in the literature.^1,9,10^ It should also be emphasized that none of the spin symmetric states with S = 1, 3, 4 are found to be stable in the unrestricted DFT regime.

DFT geometry optimized complexes for all spin states again preferred singled BS as the ground state. BLYP leads to the shorter Cr^I^–Cr^I^ bond length (covalent interaction regime), whereas B3LYP leads to a longer bond length (antiferromagnetic interaction regime), which agrees with previous studies.^26^ The dependence of bonding parameters (MBO, EBO, CSC) on the distance between the chromium atoms confirms the covalent character of the first minimum (shorter Cr^I^–Cr^I^ distance of ∼1.7 Å) and the antiferromagnetic character at a longer Cr^I^–Cr^I^ distance of ∼2.5 Å, having an S-bend shaped curve. Herein, the geometry optimizations at the restricted and unrestricted BS singlet BLYP levels of theory did not converge to the same curve as found for a symmetric system with a diazene ligand (Ar^N^CrCrAr^N^),^26^ and we find the restricted singlet geometry to be an unstable state.

Hence, we find the pure GGA (type I) DFT functionals well matching the CASSCF/NEVPT2 results when considering the spin state energetics, the electronic structure (Cr^I^–Cr^I^ bond order), the strong antiferromagnetic coupling in the ground state and optimized geometries for metal–metal complexes of a singlet spin state nature. In addition, the CASSCF QTAIM delocalization index with a value of 18 shows the need of a proper treatment of two-electron reduced density matrices making this system to be accounted for in future benchmark studies.

## Supplementary Material



## Data Availability

All data supporting the conclusion can be found in the Supporting Information, and additional information are available upon request.

## Notes

List of abbreviations; total energy for different methods (Table S1); relative energies of state-specific and state-averaged CAS­(10,10) and NEVPT2 calculations (Table S2); single-point energetics, Mulliken charges and spins of **S** and SIYNAQ for different DFT calculation settings: functional choice, basis set choice, relativity, compound size (Tables S3–S14); CAS­(10,10) composition of lowest roots of each spin state of **S** (Table S15); CAS­(10,15) natural orbitals (Figure S1); QTAIM parameters of **S** for UB3LYP and UBLYP/def2-SVP methods (Tables S16–S19 and Figures S2–S4); spin density of **S** (Figure S5); bond distances, energetics, Mulliken charges and spins of geometry optimized **S** DFT/def2-SVP (Tables S20–S26); optimized geometry of **S** by UB3LYP, UBLYP, CAS­(10,10)/def2-SVP methods (Figures S6–S8); CAS­(10,10) composition of lowest roots of each spin state of **S** for *d*_Cr–Cr_ = 2.5 Å (Table S27); composition of natural orbitals for CAS­(10,10) of S in experimental geometry and at *d*_Cr–Cr_ = 1.7 and 2.5 Å (Table S28); PEC (around minima) of all spin state of CAS­(10,10), NEVPT2 and DFT methods (Figure S9); and PEC of diamidinate Cr^I^–Cr^*I*^ (Huang et al.) for different settings (Figure S10) (PDF)
