# Supplementary material for: Cr(I)–Cr(I) Terphenyl Bridged Complexes: A Broken-Symmetry DFT and Multideterminant CASSCF/NEVPT2 Handshake
Source: ACS Omega. 2025 Oct 8;10(41):48652–61. doi: 10.1021/acsomega.5c06364 (PMC12547557; doi:10.1021/acsomega.5c06364)
Supplement: Supplementary file 1 [file ao5c06364_si_001.pdf]

Cr(I)-Cr(I) terphenyl bridged complexes: A broken symmetry DFT and multideterminant CASSCF/NEVPT2 handshake

Supporting information

Andrej Hlinčík[1], Michal Malček[1], Karol Lušpai[1], Jozef Kožíšek[1], Lukas Bucinsky[1]

[1]Institute of Physical Chemistry and Chemical Physics, Faculty of Chemical and Food Technology, Slovak University of Technology in Bratislava, Radlinského 9, SK-812 37, Bratislava, Slovak Republic

Correspondence: Lukas Bucinsky (lukas.bucinsky@stuba.sk)

Contents

List of abbreviations……………………..………………………..……………..……………………...………3

Single point calculations...4

Table S1. Total energy E of the ground state (S = 0) of SIYNAQ and **S** for different methods 4

Table S2. Relative energies [kJ.mol^{-1}] of five lowest roots for each spin state for CAS(10,10) and NEVPT2 calculations comparing state-specific (SS) and state-averaged (SA) calculations (def2-SVP basis set). Total energy [hartree] of the ground state (lowest singlet state) is given on the last line. 5

Table S3. UB3LYP/def2-SVP single point calculations of **S** with relative energies, and Mulliken charges (q) and spins (spin) of Cr atoms 6

Table S4. UBLYP/def2-SVP single point calculations of **S** with relative energies, and Mulliken charges (q) and spins (spin) of Cr atoms 6

Table S5. DKH2/UB3LYP/jorge-DZP-DKH single point calculations of **S** with relative energies, and Mulliken charges (q) and spins (spin) of Cr atoms...7

Table S6. DKH2/UBLYP/jorge-DZP-DKH single point calculations of **S** with relative energies, and Mulliken charges (q) and spins (spin) of Cr atoms...7

Table S7. DKH2 relative energies (ΔE in kJ mol^{-1}) of **S** for UBLYP, UB3LYP, CAS(10, 10) and NEVPT2 methods using the jorge-DZP-DKH basis set 7

Table S8. UB3LYP/def2-SVP single point calculations of SIYNAQ with relative energies, and Mulliken charges (q) and spins (spin)..8

Table S9. UBLYP/def2-SVP single point calculations of SIYNAQ with relative energies, and Mulliken charges (q) and spins (spin)..8

Table S10. UB3LYP/def2-TZVPP single point calculations of **S** with relative energies, and Mulliken charges (q) and spins (spin) 8

Table S11. UBLYP/def2-TZVPP single point calculations of **S** with relative energies, and Mulliken charges (q) and spins (spin) 9

Table S12. UωB97X-D/def2-SVP single point calculations of **S** with relative energies, and Mulliken charges (q) and spins (spin) 9

Table S13. UM06-2X/def2-SVP single point calculations of **S** with relative energies, and Mulliken charges (q) and spins (spin) 9

Table S14. UB3LYP-GD3/def2-SVP single point calculations of **S** with relative energies, and Mulliken charges (q) and spins (spin)...10

Table S15. BS singlet BLYP determinant based on molecular orbitals and five main configurations of each spin state of CAS(10,10) S =0 state-specific calculation of **S** with experimental geometry d_{Cr-Cr} = 1.807598 Å..........11

Table S16. QTAIM charges and spins on Cr atoms for UBLYP and UB3LYP methods of **S**, with the def2-SVP basis set 12
Table S17. Delocalization indexes between Cr-Cr and Cr-C atoms for **S** 12
Table S18. UB3LYP/def2-SVP QTAIM electron densities in the bond critical point ρBCP[bohr^{-3}], bond critical point Laplacians $\nabla 2\rho BCP$[bohr^{-5}] and ellipticities ε[-] for **S** 13
Table S19. UBLYP/def2-SVP QTAIM electron densities in the bond critical point ρBCP[bohr^{-3}], bond critical point Laplacians $\nabla 2\rho BCP$[bohr^{-5}] and ellipticities ε[-] for **S** 13
Figure S1. CAS(10,15)/def2-SVP S = 0 natural orbitals and occupation numbers (d character) of complex **S** 14
Figure S2. QTAIM parameters for different multiplicities for experimental structure of Cr(1)-Cr(2) bond for **S** at BLYP/def2-SVP and B3LYP/def2-SVP level (rho=ρBCP, lapl=$\nabla 2\rho BCP$, eli= ε) 15
Figure S3. QTAIM parameters for different multiplicities for experimental structure of Cr(1)-C(1) bond for **S** at BLYP/def2-SVP and B3LYP/def2-SVP level (rho=ρBCP, lapl=$\nabla 2\rho BCP$, eli= ε) 15
Figure S4.QTAIM parameters for different multiplicities for experimental structure of Cr(1)-C(7)^{i} bond for **S** at BLYP/def2-SVP and B3LYP/def2-SVP level (rho=ρBCP, lapl=$\nabla 2\rho BCP$, eli= ε) 16
Figure S5. UBLYP/def2-SVP spin density of **S** in BS spin state S = 0 16
Geometry optimizations........17
Table S20. U3BLYP/def2-SVP bond distances in Å after geometry optimization of **S** between chosen atoms....17
Table S21. UBLYP/def2-SVP bond distances in Å after geometry optimization of **S** between chosen atoms......17
Table S22. Dihedral angles between chosen atoms and Mulliken spins on chromium atoms of **S** for different optimization methods 18
Table S23. UB3LYP/def2-SVP relative energies, S^2, Mulliken charges and spins on chromium atoms after geometry optimization of **S** 18
Table S24. UBLYP/def2-SVP relative energies, S^2, Mulliken charges and spins on chromium atoms after geometry optimization of **S** 18
Table S25. ωB97X-D, M06-2X, and B3LYP-GD3 def2-SVP bond distances in Å after geometry optimization of **S** between chosen atoms for singlet spin states only 19
Table S26. UBLYP/def2-SVP relative energies, S^2, Mulliken charges and spins on chromium atoms after geometry optimization of **S** for the singlet spin states 19
Figure S6. Optimized geometries of **S** for different spin states by UB3LYP/def2-SVP method [1] 20
Figure S7. Optimized geometries of **S** for different spin states by UBLYP/def2-SVP method [1] 21
Figure S8. CAS(10, 10) optimized geometries of **S** for different frozen Cr-Cr distances [Å] 22
Table S27. Five main configurations of each spin state of CAS(10,10) S = 0 state-specific calculation of the optimized **S** complex at a Cr^{I}- Cr^{I} distance equal to 2.5 Å 23
Table S28. Composition of natural orbitals active space of CAS(10,10) S = 0 state-specific of **S** at ^{a}experimental geometry and optimized geometries of distances 1.7 Å and 2.5 Å between Cr(1)-Cr(2)........24
Figure S9. CAS(10,10) and NEVPT2 state-specific S = 0 potential energy curves and BLYP and B3LYP scans around respective equilibria of **S**, BS = broken-symmetry, R = restricted 26
Figure S10. Comparison of PEC of the diamidinate Cr^{I}-Cr^{I} system of Huang et al. [2], NEVPT2 represents calculation at (10,10) active space and def2-SVP basis set (A) and def2-TZVPP basis set (B), RASPT2-E is the diamidinate Cr^{I}-Cr^{I} calculation with an (12,22) active space and the ANO-RCC basis set (including two h functions for Cr atoms) of Huang et al. [2] 27

List of abbreviations

AO	Atomic Orbital
BCP	Bond Critical Point
BS	Broken-Symmetry
CASSCF / CAS	Complete Active Space Self-Consistent Field
CS	Closed-Shell
CSC	Closed-Shell Character
DI	Delocalization Index
DKH	Douglas-Kroll-Hess
EBO	Effective Bond Order
MBO	Mayer Bond Order
M-M	Metal-Metal
MO	Molecular Orbital
NEVPT2	N Electron Valence Perturbation Theory
NR	Non-Relativistic
PEC	Potential Energy Curve
QTAIM	Quantum Theory of Atoms In Molecules
RI	Resolution of Identity approximation
S	Spin quantum number
WBO	Wiberg Bond Order

Single point calculations

Table S1. Total energy E of the ground state (S = 0) of SIYNAQ and **S** for different methods

Method	System	E / a.u.
UB3LYP/def2-SVP	SIYNAQ	-5233.51932
UBLYP/def2-SVP	SIYNAQ	-5231.93934
UB3LYP/def2-SVP	**S**	-3475.15225
UBLYP/def2-SVP	**S**	-3474.60327
UB3LYP/def2-TZVPP	**S**	-3476.92465
ωB97X-D/def2-SVP	**S**	-3474.67536
M06-2X/def2-SVP	**S**	-3474.42408
B3LYP-GD3/def2-SVP	**S**	-3475.23896
UBLYP/def2-TZVPP	**S**	-3476.41138
UB3LYP/jorge-DZP-DKH	**S**	-3489.68785
UBLYP/jorge-DZP-DKH	**S**	-3489.16604
CAS (S = 5 state-specific reference)/def2-SVP	**S**	-3463.64706
NEVPT2 (S = 5 state-specific reference)/def2-SVP	**S**	-3468.94741
CAS (S = 0 state-specific reference)/def2-SVP	**S**	-3463.68465
NEVPT2 (S = 0 state-specific reference)/def2-SVP	**S**	-3468.96458

Table S2. Relative energies [kJ mol^{-1}] of five lowest roots for each spin state for CAS(10,10) and NEVPT2 calculations comparing state-specific (SS) and state-averaged (SA) calculations (def2-SVP basis set). Total energy [hartree] of the ground state (lowest singlet state) is given on the last line.

		SS singlet S[0]		SA singlet 30 S[0] R[30]		SA[1] S[3,2,1,0] R[1,14,4,5]		SA – bweight[2] S[3,2,1,0] R[1,14,4,5]		SA singlet 300 S[0] R[300]	
S	root	CASSCF	NEVPT2	CASSCF	NEVPT2	CASSCF	NEVPT2	CASSCF	NEVPT2	CASSCF	NEVPT2
5	1	771.8	793.0	706.7	781.8	650.4	759.8	694.5	775.6	598.8	742.0
4	1	574.1	576.5	522.5	566.8	479.5	548.2	512.9	561.3	442.4	535.0
	2	727.4	653.8	662.0	638.3	645.3	634.6	667.8	640.8	607.5	623.5
	3	750.1	676.9	683.0	659.2	666.8	655.6	689.3	662.1	628.0	642.5
	4	779.6	703.7	721.7	699.0	683.7	680.9	716.1	692.6	644.6	670.0
	5	787.9	705.5	736.4	702.8	689.3	683.3	724.0	695.4	650.1	672.3
3	1	373.5	364.3	334.1	353.4	307.9	341.4	329.1	350.3	285.0	333.1
	2	530.6	455.8	484.5	433.3	473.4	428.8	488.1	435.7	450.4	419.4
	3	535.3	452.5	488.8	443.0	479.3	440.3	492.0	444.3	456.7	432.8
	4	570.0	494.0	529.7	484.5	501.2	471.8	522.6	481.0	476.6	463.2
	5	592.2	516.1	543.0	501.0	531.4	496.9	546.6	503.3	505.3	488.1
2	1	183.7	171.4	156.7	159.1	146.8	154.2	155.9	158.6	137.0	151.0
	2	392.9	321.6	372.5	316.9	368.0	321.4	373.5	317.9	356.3	326.9
	3	422.3	358.4	398.0	346.9	393.0	343.7	400.9	348.2	382.0	328.7
	4	426.8	372.4	401.9	354.7	396.1	346.3	401.8	355.2	384.9	344.9
	5	441.8	376.8	417.0	366.5	414.8	366.2	420.1	367.5	399.9	365.9
1	1	67.5	66.0	56.7	60.2	52.8	57.4	56.4	59.7	49.4	56.2
	2	224.3	176.3	207.8	177.7	211.4	177.5	211.6	179.1	205.5	173.6
	3	226.2	188.9	208.8	169.4	213.3	166.4	212.6	169.3	207.6	165.1
	4	279.3	230.5	258.7	217.4	265.9	220.3	264.5	220.8	256.8	213.6
	5	310.4	254.4	278.9	241.1	277.1	235.6	281.9	240.6	267.7	233.8
0	1	0.0	0.0	0.0	0.0	0.0	0.0	0.0	0.0	0.0	0.0
	2	300.8	240.7	285.2	231.9	294.2	230.5	291.1	232.7	287.4	227.6
	3	326.6	282.0	300.7	265.8	303.5	263.7	305.2	267.4	293.6	257.4
	4	348.8	278.9	329.8	266.2	340.5	267.7	337.0	269.0	331.5	261.7
	5	350.7	286.3	330.5	272.1	341.9	275.8	338.2	276.3	332.2	268.0
0	1	-3463.683	-3468.962	-3463.679	-3468.965	-3463.679	-3468.961	-3463.681	-3468.963	-3463.671	-3468.966

[1] SA calculations including spin states S=[3,2,1,0] and the number of roots=[1,14,4,5] with weighting all spin states equally; [2] SA calculations including spin states S=[3,2,1,0] and the number of roots=[1,14,4,5] with weighting all spin states according to the number of roots

Table S3. UB3LYP/def2-SVP single point calculations of **S** with relative energies, and Mulliken charges (q) and spins (spin) of Cr atoms

S	ΔE / kJ mol^{-1}	S^2	q(Cr(1))	q(Cr(2))	spin(Cr(1))	spin(Cr(2))
**0**	**0.00**	**2.726**	**0.186**	**0.186**	**3.474**	**-3.474**
0[a]	190.97		0.150	0.150		
1	103.94	3.418	0.197	0.176	3.162	-1.336
1	159.05	2.018	0.147	0.147	0.956	0.956
2	154.64	6.020	0.198	0.198	1.906	1.906
3	299.25	12.039	0.195	0.188	2.912	2.905
3	302.58	12.054	0.191	0.191	2.916	2.916
4	465.88	20.038	0.182	0.205	3.875	3.928
4	466.24	20.037	0.194	0.194	3.889	3.889
5	626.28	30.044	0.228	0.228	4.867	4.867

[a] RBLYP calculation; preferred spin state marked by bold; red color represents unstable state

Table S4. UBLYP/def2-SVP single point calculations of **S** with relative energies, and Mulliken charges (q) and spins (spin) of Cr atoms

S	ΔE / kJ mol^{-1}	S^2	q(Cr(1))	q(Cr(2))	spin(Cr(1))	spin(Cr(2))
**0**	**0.00**	**1.379**	**0.106**	**0.106**	**2.213**	**-2.213**
0[a]	26.82	-	0.095	0.095	-	-
1	66.87	2.196	0.106	0.097	1.520	0.193
1	67.45	2.009	0.097	0.097	0.861	0.861
2	118.34	6.013	0.140	0.139	1.785	1.785
3	335.69	12.026	0.134	0.129	2.750	2.787
3	337.47	12.029	0.129	0.129	2.775	2.775
4	576.93	20.031	0.134	0.148	3.701	3.755
5	805.05	30.037	0.169	0.169	4.687	4.688

[a] RBLYP calculation; preferred spin state marked by bold; red color represents unstable state

Table S5. DKH2/UB3LYP/jorge-DZP-DKH single point calculations of **S** with relative energies, and Mulliken charges (q) and spins (spin) of Cr atoms

S	ΔE / kJ mol^{-1}	S^2	q(Cr(1))	q(Cr(2))	spin(Cr(1))	spin(Cr(2))
0	0.00	2.612	0.263	0.263	3.404	-3.404
0[a]	169.95	-	0.225	0.225	-	-
1	100.11	3.306	0.269	0.258	3.055	-1.273
2	151.06	6.020	0.290	0.290	1.882	1.881
3	305.38	12.036	0.297	0.275	2.880	2.879
4	479.22	20.037	0.285	0.308	3.846	3.864
5	646.59	30.045	0.315	0.314	4.821	4.821

[a] RBLYP calculation

Table S6. DKH2/UBLYP/jorge-DZP-DKH single point calculations of **S** with relative energies, and Mulliken charges (q) and spins (spin) of Cr atoms

S	ΔE / kJ mol^{-1}	S^2	q(Cr(1))	q(Cr(2))	spin(Cr(1))	spin(Cr(2))
0	0.00	1.248	0.210	0.210	2.088	-2.088
0[a]	21.20	-	0.198	0.198	-	-
1	66.10	2.111	0.201	0.198	1.315	0.345
2	123.31	6.013	0.259	0.259	1.762	1.762
3	347.33	12.024	0.263	0.256	2.698	2.751
4	592.92	20.031	0.272	0.284	3.662	3.673
5	826.87	30.037	0.289	0.288	4.634	4.635

[a] RBLYP calculation

Table S7. DKH2 relative energies (ΔE in kJ mol^{-1}) of **S** for UBLYP, UB3LYP, CAS(10, 10) and NEVPT2 methods using the jorge-DZP-DKH basis set

			S = 5 (state-specific)		S = 0 (state-specific)	
S	UB3LYP	UBLYP	CAS(10, 10)	NEVPT2	CAS(10, 10)	NEVPT2
0	0.00	0.00	0.00	0.00	0.00	0.00
1	100.11	66.10	43.52	55.27	74.64	70.49
2	151.06	123.31	120.73	151.49	201.91	179.13
3	305.38	347.33	238.62	325.55	406.90	381.23
4	462.42	592.92	364.68	517.53	621.71	602.33
5	646.59	826.87	490.66	715.43	831.78	827.98

Table S8. UB3LYP/def2-SVP single point calculations of SIYNAQ with relative energies, and Mulliken charges (q) and spins (spin)

S	ΔE / kJ mol-1	S2	q(Cr(1))	q(Cr(2))	spin(Cr(1))	spin(Cr(2))
0	0.00	2.714	0.162	0.162	-3.431	3.431
0[a]	188.80	-	0.123	0.123	-	-
1	104.30	3.408	0.150	0.174	-1.282	3.116
2	153.08	6.020	0.174	0.174	1.910	1.910
3	297.73	12.041	0.164	0.171	2.912	2.912
4	467.61	20.040	0.153	0.182	3.888	3.940
5	630.58	30.045	0.198	0.198	4.880	4.880

[a] RBLYP calculation

Table S9. UBLYP/def2-SVP single point calculations of SIYNAQ with relative energies, and Mulliken charges (q) and spins (spin)

S	ΔE / kJ mol-1	S2	q(Cr(1))	q(Cr(2))	spin(Cr(1))	spin(Cr(2))
0	0.00	1.380	0.071	0.071	-2.186	2.186
0[a]	26.63	-	0.057	0.057	-	-
1	68.50	2.234	0.060	0.071	0.153	1.564
2	117.76	6.013	0.102	0.102	1.794	1.794
3	333.06	12.027	0.092	0.097	2.788	2.759
4	577.29	20.032	0.092	0.109	3.700	3.755
5	806.51	30.038	0.128	0.128	4.677	4.677

[a] RBLYP calculation

Table S10. UB3LYP/def2-TZVPP single point calculations of **S** with relative energies, and Mulliken charges (q) and spins (spin)

S	ΔE / kJ mol-1	S2	q(Cr(1))	q(Cr(2))	spin(Cr(1))	spin(Cr(2))
0	0.00	2.686	0.148	0.148	3.816	-3.816
0[a]	180.47	-	0.108	0.108	-	-
1	100.98	3.389	0.159	0.133	3.456	-1.616
2	152.77	6.021	0.137	0.137	1.892	1.892
3	293.62	12.039	0.147	0.134	2.916	2.910
4	462.42	20.037	0.153	0.153	3.890	3.896
5	623.79	30.045	0.167	0.167	4.899	4.899

[a] RBLYP calculation

Table S11. UBLYP/def2-TZVPP single point calculations of **S** with relative energies, and Mulliken charges (q) and spins (spin)

S	ΔE / kJ mol^{-1}	S^2	q(Cr(1))	q(Cr(2))	spin(Cr(1))	spin(Cr(2))
0	0.00	1.312	0.100	0.100	2.441	-2.441
0[a]	23.53	-	0.092	0.091	-	-
1	66.23	2.172	0.107	0.099	1.609	0.128
2	119.49	6.013	0.112	0.111	1.774	1.774
3	331.27	12.026	0.096	0.126	2.753	2.795
4	570.18	20.033	0.120	0.118	3.682	3.729
5	796.84	30.038	0.114	0.114	4.709	4.710

[a] RBLYP calculation

Table S12. UωB97X-D/def2-SVP single point calculations of **S** with relative energies, and Mulliken charges (q) and spins (spin)

S	ΔE / kJ mol^{-1}	S^2	q(Cr(1))	q(Cr(2))	spin(Cr(1))	spin(Cr(2))
0	0.00	2.991	0.244	0.244	3.608	-3.608
0[a,b]	262.81	-	0.205	0.205	-	-
1	111.78	3.726	0.258	0.235	3.387	-1.545
1[b]	194.59	2.516	0.212	0.201	0.626	1.297
2	173.87	6.295	0.270	0.237	2.775	1.003
3	300.16	12.039	0.250	0.242	2.899	2.885
4	455.68	20.030	0.232	0.256	3.854	3.900
5	607.07	30.032	0.275	0.275	4.843	4.843

[a] RBLYP calculation, [b] unstable state

Table S13. UM06-2X/def2-SVP single point calculations of **S** with relative energies, and Mulliken charges (q) and spins (spin)

S	ΔE / kJ mol^{-1}	S^2	q(Cr(1))	q(Cr(2))	spin(Cr(1))	spin(Cr(2))
0	0.00	3.785	0.322	0.322	4.264	-4.264
0[a,b]	511.56	-	0.269	0.269	-	-
1	151.37	4.758	0.404	0.218	4.173	-2.268
1[b]	417.20	2.452	0.258	0.253	1.009	0.991
2	238.67	7.515	0.274	0.359	-0.149	4.046
3	354.63	12.541	0.342	0.273	4.034	1.904
4	409.88	20.073	0.292	0.325	3.980	4.009
5	457.96	30.038	0.330	0.330	4.956	4.956

[a] RBLYP calculation, [b] unstable state

Table S14. UB3LYP-GD3/def2-SVP single point calculations of **S** with relative energies, and Mulliken charges (q) and spins (spin)

S	ΔE / kJ mol^{-1}	S^2	q(Cr(1))	q(Cr(2))	spin(Cr(1))	spin(Cr(2))
0	0.00	2.727	0.186	0.186	3.475	-3.475
0[a]	191.01	-	0.150	0.150	-	-
1	103.98	3.418	0.197	0.176	3.162	-1.336
1[b]	159.10	2.018	0.147	0.147	0.956	0.956
2	154.68	6.020	0.198	0.198	1.906	1.906
3	299.29	12.039	0.195	0.188	2.912	2.905
4	465.93	20.038	0.182	0.205	3.875	3.928
5	626.32	30.044	0.228	0.228	4.867	4.867

[a] RBLYP calculation, [b] unstable state

Table S15. BS singlet BLYP determinant based on molecular orbitals and five main configurations of each spin state of CAS(10,10) S =0 state-specific calculation of **S** with experimental geometry $d_{Cr\text{-}Cr}$ = 1.807598 Å

Method	S	configuration	weight / %	$\Delta E_{CAS(10,10)}$/ kJ mol^{-1}
[a] BS UBLYP	0	$\pi_{yz}^2\pi_{xz}^2\sigma_{z^2}^2(\delta_{x^2-y^2}^{1n})^{\alpha}(\delta_{x^2-y^2}^{1n})^{\beta}(\delta_{xy}^{1n})^{\alpha}(\delta_{xy}^{1n})^{\beta}$	-	-
CAS(10,10)	0	$1\sigma^2 1\pi^2 2\pi^2 1\delta^2 2\delta^2$	42.5	0.00
		$1\sigma^2 1\pi^2 2\pi^2 1\delta^2 2\delta^{*2}$	8.1	
		$1\sigma^2 1\pi^2 2\pi^2 1\delta^1 1\delta^{*1} 2\delta^1 2\delta^{*1}$	5.2	
		$1\sigma^2 1\pi^2 2\pi^2 1\delta^{*2} 2\delta^2$	4.1	
		$1\sigma^2 1\pi^2 2\pi^1 2\pi^{*1} 1\delta^2 2\delta^1 2\delta^{*1}$	2.6	
CAS(10,10)	1	$1\sigma^2 1\pi^2 2\pi^2 1\delta^2 2\delta^1 2\delta^{*1}$	32.0	67.34
		$1\sigma^2 1\pi^2 2\pi^2 1\delta^1 1\delta^{*1} 2\delta^2$	14.6	
		$1\sigma^2 1\pi^2 2\pi^2 1\delta^{*2} 2\delta^1 2\delta^{*1}$	4.0	
		$1\sigma^1 1\sigma^{*1} 1\pi^2 2\pi^2 1\delta^2 2\delta^1 2\delta^{*1}$	3.8	
		$1\sigma^2 1\pi^2 2\pi^1 2\pi^{*1} 1\delta^1 1\delta^{*1} 2\delta^2$	3.1	
CAS(10,10)	2	$1\sigma^2 1\pi^2 2\pi^2 1\delta^1 1\delta^{*1} 2\delta^1 2\delta^{*1}$	42.3	183.29
		$1\sigma^2 1\pi^2 2\pi^1 2\pi^{*1} 1\delta^2 2\delta^1 2\delta^{*1}$	5.1	
		$1\sigma^2 1\pi^1 1\pi^{*1} 2\pi^2 1\delta^2 2\delta^1 2\delta^{*1}$	4.2	
		$1\sigma^1 1\sigma^{*1} 1\pi^2 2\pi^2 1\delta^2 2\delta^1 2\delta^{*1}$	3.9	
		$1\sigma^2 1\pi^2 2\pi^1 2\pi^{*1} 1\delta^1 1\delta^{*1} 2\delta^2$	3.5	
CAS(10,10)	3	$1\sigma^2 1\pi^2 2\pi^1 2\pi^{*1} 1\delta^1 1\delta^{*1} 2\delta^1 2\delta^{*1}$	21.1	372.66
		$1\sigma^2 1\pi^1 1\pi^{*1} 2\pi^2 1\delta^1 1\delta^{*1} 2\delta^1 2\delta^{*1}$	18.5	
		$1\sigma^1 1\sigma^{*1} 1\pi^2 2\pi^2 1\delta^1 1\delta^{*1} 2\delta^1 2\delta^{*1}$	15.1	
		$1\sigma^1 1\sigma^{*1} 1\pi^1 1\pi^{*1} 2\pi^1 2\pi^{*1} 1\delta^1 1\delta^{*1} 2\delta^1 2\delta^{*1}$	4.1	
		$1\sigma^2 1\pi^1 1\pi^{*1} 2\pi^1 2\pi^{*1} 1\delta^2 2\delta^1 2\delta^{*1}$	3.8	
CAS(10,10)	4	$1\sigma^2 1\pi^1 1\pi^{*1} 2\pi^1 2\pi^{*1} 1\delta^1 1\delta^{*1} 2\delta^1 2\delta^{*1}$	29.0	572.87
		$1\sigma^1 1\sigma^{*1} 1\pi^2 2\pi^1 2\pi^{*1} 1\delta^1 1\delta^{*1} 2\delta^1 2\delta^{*1}$	25.0	
		$1\sigma^1 1\sigma^{*1} 1\pi^1 1\pi^{*1} 2\pi^2 1\delta^1 1\delta^{*1} 2\delta^1 2\delta^{*1}$	17.8	
		$1\sigma^1 1\sigma^{*1} 1\pi^1 1\pi^{*1} 2\pi^1 2\pi^{*1} 1\delta^2 2\delta^1 2\delta^{*1}$	6.3	
		$1\sigma^1 1\sigma^{*1} 1\pi^1 1\pi^{*1} 2\pi^1 2\pi^{*1} 1\delta^1 1\delta^{*1} 2\delta^2$	5.0	
CAS(10,10)	5	$1\sigma^1 1\sigma^{*1} 1\pi^1 1\pi^{*1} 2\pi^1 2\pi^{*1} 1\delta^1 1\delta^{*1} 2\delta^1 2\delta^{*1}$	100.0	770.12

[a] n represents a non-bonding orbital

Table S16. QTAIM charges and spins on Cr atoms for UBLYP and UB3LYP methods of **S**, with the def2-SVP basis set

S	UB3LYP charge		UB3LYP spin		UBLYP charge		UBLYP spin	
	Cr(1)	Cr(2)	Cr(1)	Cr(2)	Cr(1)	Cr(2)	Cr(1)	Cr(2)
**0**	**0.657**	**0.657**	**3.127**	**-3.127**	**0.642**	**0.642**	**1.983**	**-1.983**
0[a]	0.645	0.645	0.000	0.000	0.636	0.636	0.000	0.000
1	0.668	0.658	2.855	-1.152	0.643	0.641	1.374	0.231
1	0.637	0.637	0.901	0.901	0.639	0.639	0.808	0.808
2	0.667	0.667	1.767	1.767	0.677	0.677	1.643	1.643
3	0.668	0.665	2.748	2.739	0.675	0.673	2.585	2.618
3	0.663	0.663	2.754	2.754	0.669	0.669	2.609	2.609
4	0.691	0.683	3.660	3.741	0.707	0.699	3.497	3.566
4	0.689	0.689	3.688	3.688	-	-	-	-
5	0.724	0.724	4.587	4.587	0.734	0.734	4.417	4.417
0[b]	0.683	0.683	3.077	-3.077	0.673	0.672	1.912	-1.912

[a] RBLYP calculation; preferred spin state marked by bold; red color represents unstable state,

[b] def2-TZVPP basis set

Table S17. Delocalization indexes between Cr-Cr and Cr-C atoms for **S**

S	UB3LYP/def2-SVP			UBLYP/def2-SVP		
	Cr(1)-Cr(2)	Cr(1)-C(1)	Cr(1)-C(7)[i]	Cr(1)-Cr(2)	Cr(1)-C(1)	Cr(1)-C(7)[i]
**0**	**2.2694**	**0.6639**	**0.2170**	**3.1463**	**0.7022**	**0.2265**
0[a]	3.9800	0.6980	0.2150	3.9497	0.7119	0.2214
1	2.5031	0.6626	0.2166	3.3144	0.7058	0.2263
1	3.4389	0.6807	0.2178	3.4289	0.7091	0.2267
2	2.7948	0.6698	0.2278	2.8121	0.7068	0.2485
3	2.0496	0.6730	0.2337	2.0868	0.7114	0.2572
3	2.0466	0.6752	0.2302	2.0879	0.7186	0.2514
4	1.3107	0.6658	0.2443	1.3738	0.7126	0.2566
4	1.3154	0.6761	0.2404	-	-	-
5	0.6500	0.6770	0.2370	0.7200	0.7310	0.2460
0[b]	2.3052	0.6640	0.2245	3.1785	0.7018	0.2339

[a] Restricted calculation, red color represents an unstable state, [b] def2-TZVPP basis set

Table S18. UB3LYP/def2-SVP QTAIM electron densities in the bond critical point ρ_{BCP}[bohr^{-3}], bond critical point Laplacians $\nabla^2\rho_{BCP}$[bohr^{-5}] and ellipticities ε[-] for **S**

S	Cr(1)-Cr(2)			Cr(1)-C(1)			Cr(1)-C(7)[i]		
	ρ_{BCP}	$\nabla^2\rho_{BCP}$	ε	ρ_{BCP}	$\nabla^2\rho_{BCP}$	ε	ρ_{BCP}	$\nabla^2\rho_{BCP}$	ε
0	0.2087	0.9762	0.0203	0.0853	0.1927	0.0464	0.0462	0.1641	0.6223
0[a]	0.2238	0.8543	0.0079	0.0880	0.1639	0.0544	0.0473	0.1606	0.6964
1	0.2149	0.9279	0.0178	0.0856	0.1883	0.0509	0.0462	0.1638	0.6094
1	0.2237	0.8553	0.0099	0.0872	0.1707	0.0444	0.0467	0.1600	0.5646
2	0.2240	0.8665	0.0239	0.0855	0.1887	0.0599	0.0460	0.1649	0.5639
3	0.1632	1.0612	0.3686	0.0854	0.1901	0.0549	0.0467	0.1609	0.4424
3	0.1526	1.2795	0.0188	0.0853	0.1884	0.0703	0.0461	0.1643	0.4819
4	0.1571	1.1848	0.7900	0.0858	0.1910	0.0800	0.0464	0.1626	0.2983
4	0.1523	1.4696	1.4492	0.0857	0.1920	0.0588	0.0480	0.1563	0.4563
5	0.1557	1.2563	0.0121	0.0853	0.1965	0.0432	0.0488	0.1517	0.6549
0[b]	0.2047	1.1461	0.0289	0.0853	0.2041	0.0391	0.0477	0.1440	1.1909

[a] RBLYP calculation, red color represents an unstable state, [b] def2-TZVPP basis set

Table S19. UBLYP/def2-SVP QTAIM electron densities in the bond critical point ρ_{BCP}[bohr^{-3}], bond critical point Laplacians $\nabla^2\rho_{BCP}$[bohr^{-5}] and ellipticities ε[-] for **S**

S	Cr(1)-Cr(2)			Cr(1)-C(1)			Cr(1)-C(7)[i]		
	ρ_{BCP}	$\nabla^2\rho_{BCP}$	ε	ρ_{BCP}	$\nabla^2\rho_{BCP}$	ε	ρ_{BCP}	$\nabla^2\rho_{BCP}$	ε
0	0.2176	0.9060	0.0154	0.0864	0.1693	0.0421	0.0466	0.1610	0.5909
0[a]	0.2205	0.8804	0.0095	0.0874	0.1582	0.0392	0.0471	0.1596	0.6186
1	0.2205	0.8836	0.0109	0.0868	0.1645	0.0374	0.0467	0.1602	0.5785
1	0.2207	0.8808	0.0098	0.0870	0.1623	0.0350	0.0469	0.1594	0.5733
2	0.2198	0.8984	0.0566	0.0861	0.1724	0.0490	0.0467	0.1613	0.5253
3	0.1621	1.0937	0.3080	0.0862	0.1707	0.0632	0.0473	0.1571	0.4415
3	0.1530	1.2911	0.0552	0.0862	0.1690	0.0789	0.0468	0.1597	0.4807
4	0.1582	1.1874	0.8060	0.0866	0.1709	0.0855	0.0473	0.1588	0.3862
5	0.1579	1.1709	0.0123	0.0866	0.1723	0.0687	0.0489	0.1519	0.6637
0[b]	0.2115	1.0865	0.0186	0.0863	0.1829	0.0362	0.0481	0.1402	1.1054

[a] RBLYP calculation, red color represents an unstable state, [b] def2-TZVPP basis set

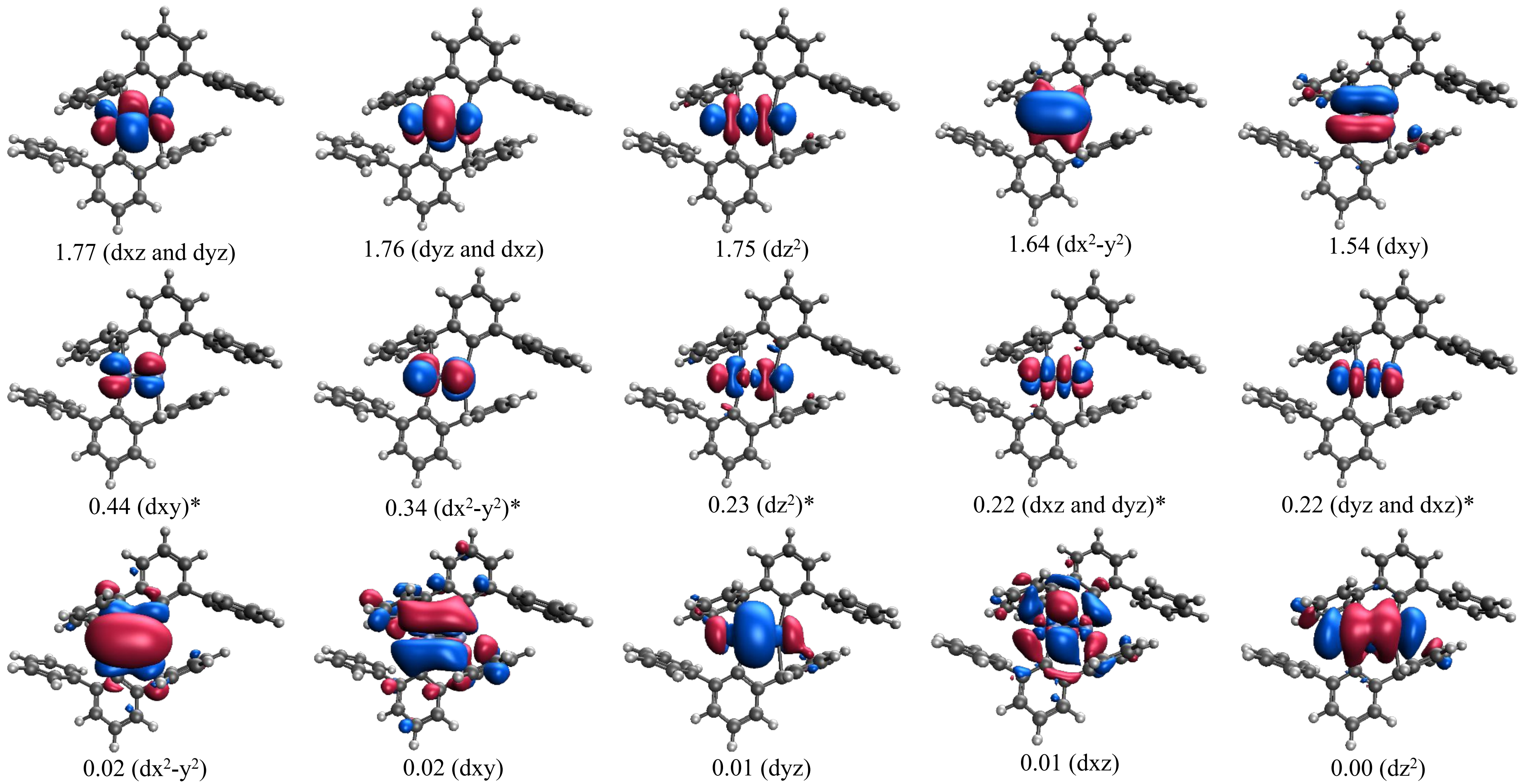

Figure S1. CAS(10,15)/def2-SVP S = 0 natural orbitals and occupation numbers (d character) of complex **S**

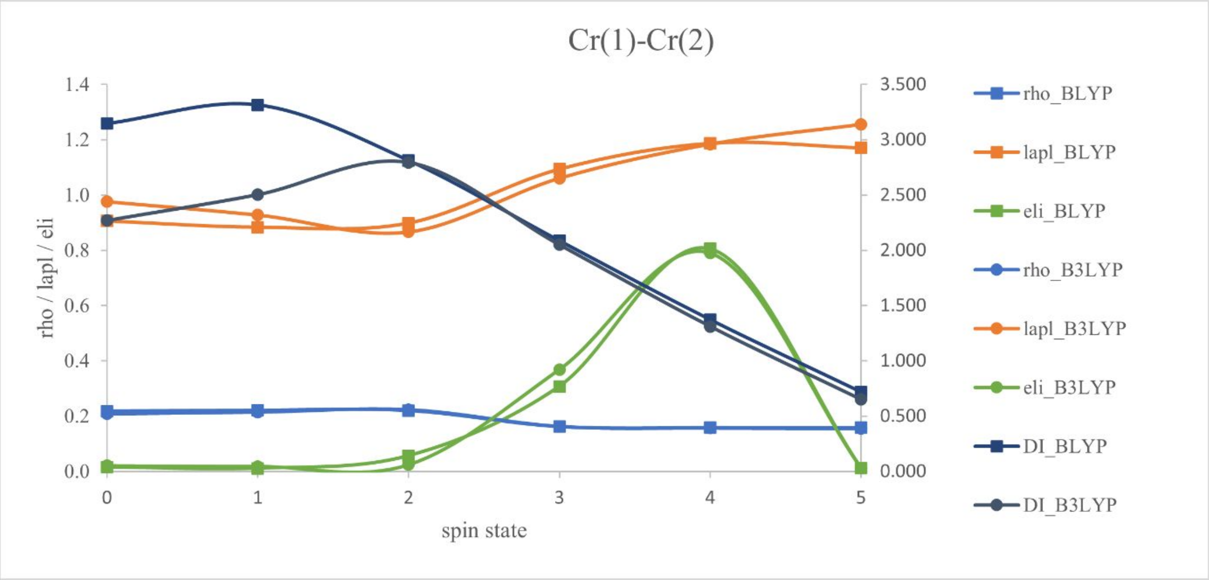

Figure S2. QTAIM parameters for different multiplicities for experimental structure of Cr(1)-Cr(2) bond for **S** at BLYP/def2-SVP and B3LYP/def2-SVP level (rho=ρ_{BCP}, lapl= $\nabla^2\rho_{BCP}$, eli= ε)

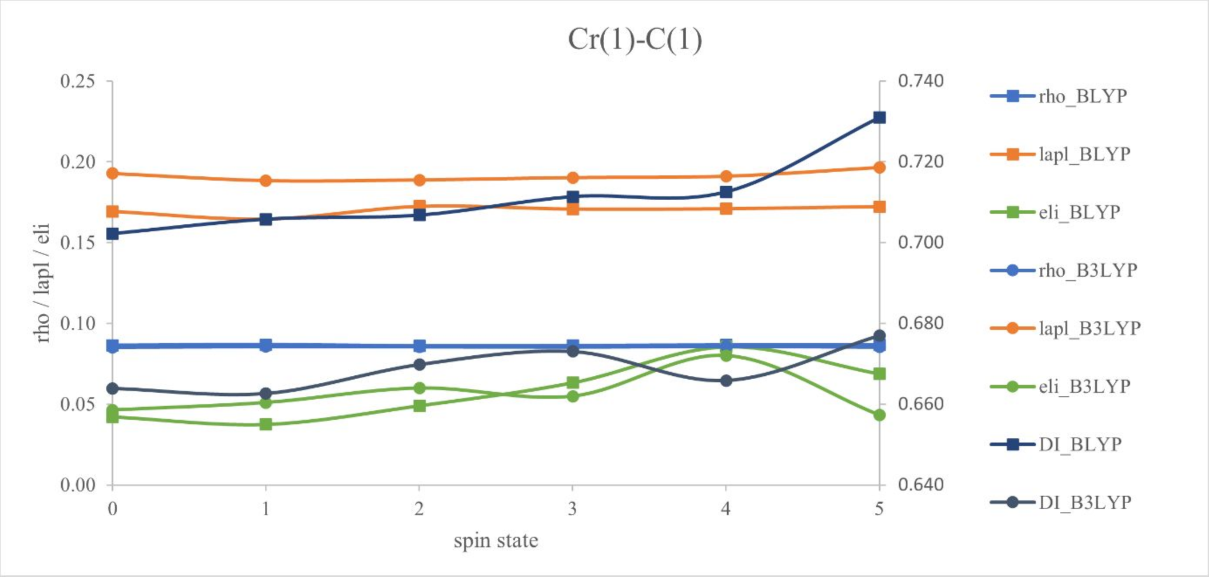

Figure S3. QTAIM parameters for different multiplicities for experimental structure of Cr(1)-C(1) bond for **S** at BLYP/def2-SVP and B3LYP/def2-SVP level (rho=ρ_{BCP}, lapl=$\nabla^2\rho_{BCP}$, eli= ε)

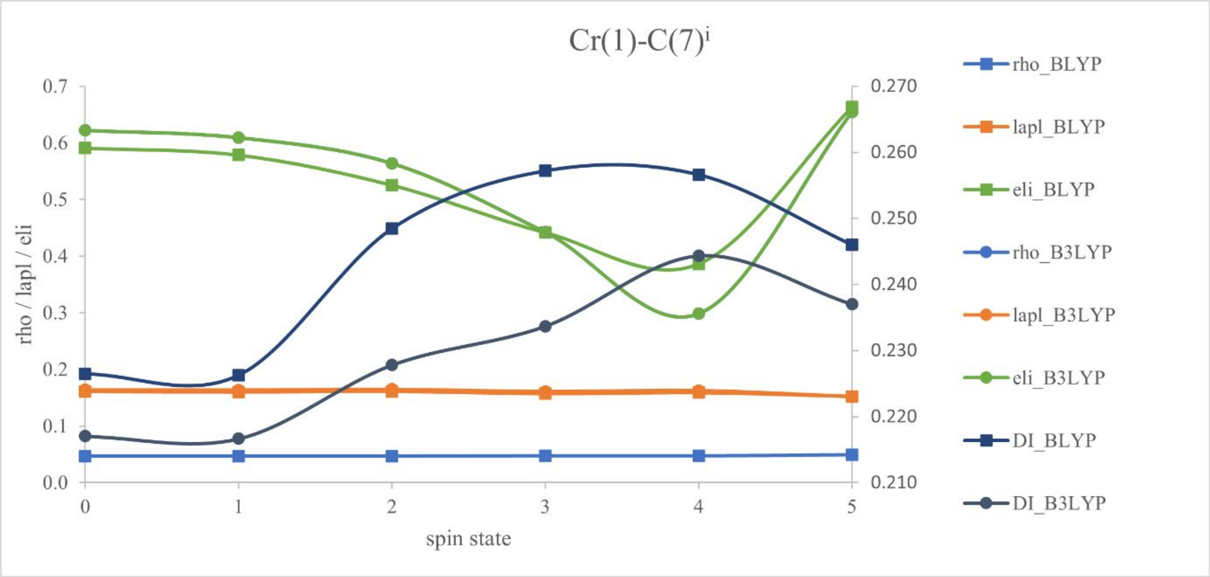

Figure S4.QTAIM parameters for different multiplicities for experimental structure of Cr(1)-C(7)^i bond for **S** at BLYP/def2-SVP and B3LYP/def2-SVP level (rho=ρ_{BCP}, lapl= $\nabla^2\rho_{BCP}$, eli= ε)

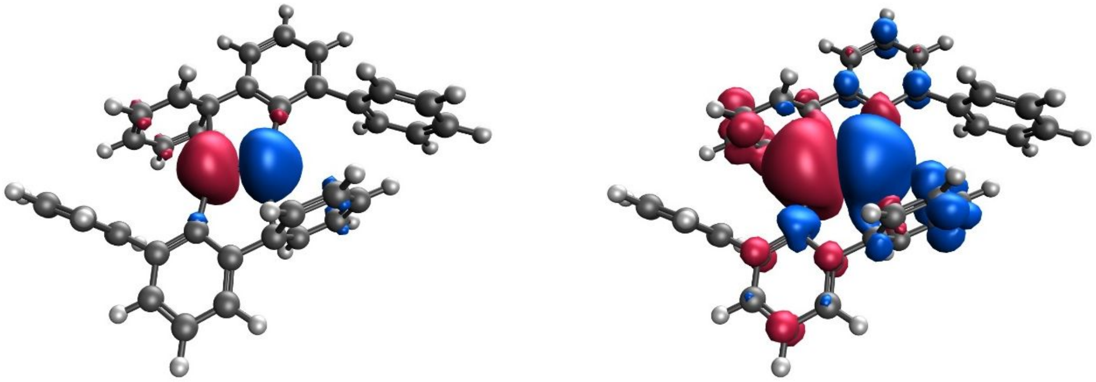

isovalue = 0.005 isovalue = 0.001

Figure S5. UBLYP/def2-SVP spin density of **S** in BS spin state S = 0

Geometry optimizations

Table S20. U3BLYP/def2-SVP bond distances in Å after geometry optimization of **S** between chosen atoms

S	Cr(1)-Cr(2)	Cr(1)-C(1)	Cr(2)-C(1)[i]	Cr(1)-C(7)[i]	Cr(2)-C(7)
**S exp**	**1.807**	**2.136**	**2.136**	**2.322**	**2.322**
0	2.585	2.117	2.117	2.574	2.574
0[a]	1.601	2.115	2.108	2.775	2.361
1	2.784	2.113	2.105	3.005	2.349
2	2.584	2.129	2.090	2.204	3.893
3	2.650	2.062	2.080	2.308	2.325
4	2.702	2.155	2.070	2.244	2.737
5	4.145	2.128	2.124	2.991	3.550

[a] RBLYP case, experimental geometry of **S** is marked in bold

Table S21. UBLYP/def2-SVP bond distances in Å after geometry optimization of **S** between chosen atoms

S	Cr(1)-Cr(2)	Cr(1)-C(1)	Cr(2)-C(1)[i]	Cr(1)-C(7)[i]	Cr(2)-C(7)
**S exp**	**1.807**	**2.136**	**2.136**	**2.322**	**2.322**
0	1.718	2.113	2.117	2.426	2.458
0[a]	1.655	2.119	2.118	2.417	2.395
1	1.740	2.078	2.079	2.389	2.391
2	2.709	2.054	1.943	2.329	2.221
3	2.834	2.050	2.050	2.506	2.506
4	3.216	2.023	2.092	2.740	2.782
5	2.943	2.121	2.176	2.492	3.062

[a] RBLYP case; experimental geometry of **S** is marked in bold

Table S22. Dihedral angles between chosen atoms and Mulliken spins on chromium atoms of **S** for different optimization methods

Method	S	Dihedral angle / °		spin(Cr(1))	spin(Cr(2))
		D1	D2		
Experiment	-	179.99	1.17	-	-
UB3LYP/def2-SVP	0	-179.96	-2.85	4.699	-4.699
UB3LYP/def2-SVP	5	-169.10	5.17	4.937	4.943
UB3LYP/def2-SVP[a]	0(R)	163.37	-13.15	-	-
UBLYP/def2-SVP	0	-178.63	2.86	1.887	-1.893
UBLYP/def2-SVP	5	168.30	-14.29	4.626	4.830
UBLYP/def2-SVP	0(R)	-179.97	0.47	-	-
CAS(10,10) (min)[b]	0	173.00	52.08	-	-
NEVPT2 (min)[b]	0	-179.99	-3.36	-	-

D1 = C(1)-Cr(1)-Cr(2)-C(1)^{i}; D2 = C(1)-Cr(1)-Cr(2)-C(7)

Table S23. UB3LYP/def2-SVP relative energies, S^2, Mulliken charges and spins on chromium atoms after geometry optimization of **S**

S	ΔE / kJ mol^{-1}	S^2	q(Cr(1))	q(Cr(2))	spin(Cr(1))	spin(Cr(2))
**0**	**0.00**	**4.581**	**0.290**	**0.290**	**4.699**	**-4.699**
0[a]	224.98	-	0.201	0.263	-	-
1	48.19	5.028	0.394	0.075	4.765	-3.338
2	95.20	6.887	-0.388	0.396	-0.955	4.583
3	97.50	12.355	0.412	-0.177	4.003	2.792
4	84.54	20.320	0.098	0.442	3.902	4.505
5	45.06	30.037	0.321	0.315	4.937	4.943

[a] RBLYP case; preferred spin state marked in bold

Table S24. UBLYP/def2-SVP relative energies, S^2, Mulliken charges and spins on chromium atoms after geometry optimization of **S**

S	ΔE / kJ mol^{-1}	S^2	q(Cr(1))	q(Cr(2))	spin(Cr(1))	spin(Cr(2))
**0**	**0.00**	**1.080**	**0.175**	**0.162**	**1.887**	**-1.893**
0[a]	9.53	-	0.156	0.166	-	-
1	52.09	2.005	0.159	0.158	0.791	0.813
2	88.00	6.186	-0.019	-0.190	2.987	1.490
3	89.37	12.243	0.011	0.011	3.185	3.185
4	121.80	20.118	0.075	0.254	3.289	4.614
5	107.80	30.034	0.272	0.237	4.626	4.830

[a] RBLYP case; preferred spin state marked in bold

Table S25. ωB97X-D, M06-2X, and B3LYP-GD3 def2-SVP bond distances in Å after geometry optimization of **S** between chosen atoms for singlet spin states only

S	Cr(1)-Cr(2)	Cr(1)-C(1)	Cr(2)-C(1)[i]	Cr(1)-C(7)[i]	Cr(2)-C(7)
**S exp**	**1.807**	**2.136**	**2.136**	**2.322**	**2.322**
ωB97X-D					
0	2.595	2.119	2.119	2.483	2.483
0[a]	1.612	2.094	2.095	2.267	2.267
M06-2X					
0	2.760	2.160	2.160	2.520	2.520
0[a]	1.655	2.115	2.120	2.378	2.437
B3LYP-GD3					
0	2.563	2.108	2.108	2.577	2.577
0[a]	1.598	2.109	2.109	2.416	2.417

[a] RBLYP case, experimental geometry of **S** is marked in bold

Table S26. UBLYP/def2-SVP relative energies, S^2, Mulliken charges and spins on chromium atoms after geometry optimization of **S** for the singlet spin states

S	ΔE / kJ mol^{-1}	S^2	q(Cr(1))	q(Cr(2))	spin(Cr(1))	spin(Cr(2))
ωB97X-D						
**0**	**0.00**	**4.621**	**0.310**	**0.310**	**4.642**	**-4.642**
0[a]	251.29	-	0.231	0.232	-	-
M06-2X						
**0**	**0.00**	**4.826**	**0.382**	**0.382**	**4.868**	**-4.868**
0[a]	-	-	-	-	-	-
B3LYP-GD3						
0	0.00	4.559	0.285	0.285	4.688	-4.688
0[a]	219.71	-	0.215	0.215	-	-

[a] RBLYP case; preferred spin state marked in bold

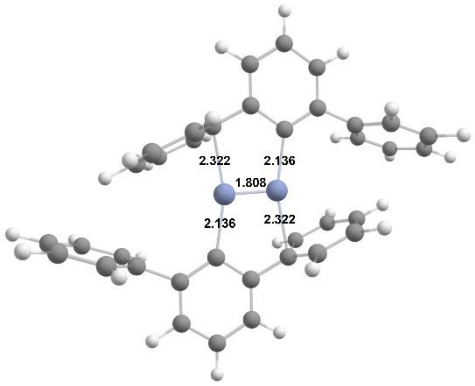

S “experimental”

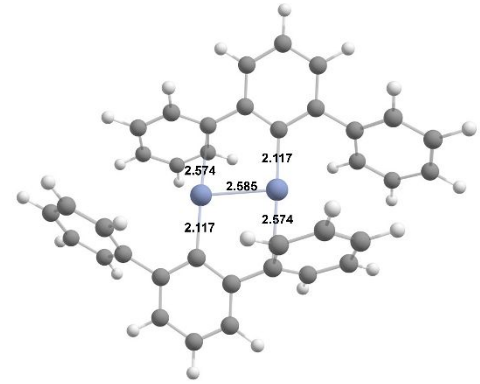

S = 0

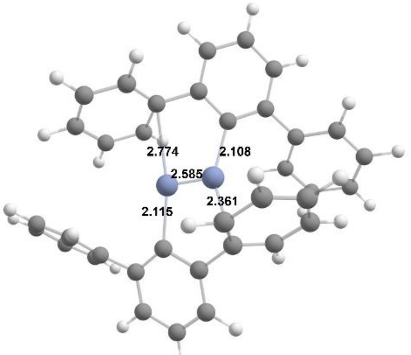

S = 0 (RBLYP)

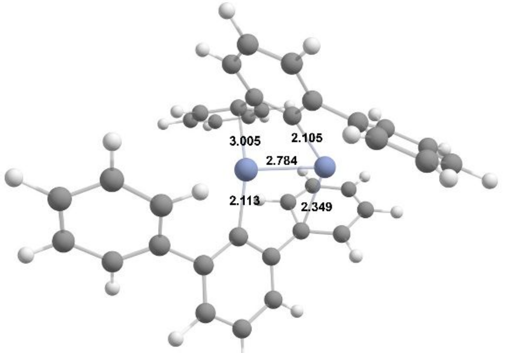

S = 1

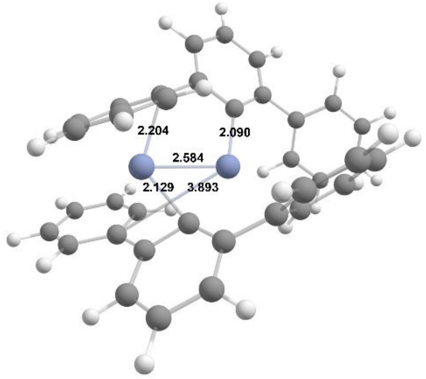

S = 2

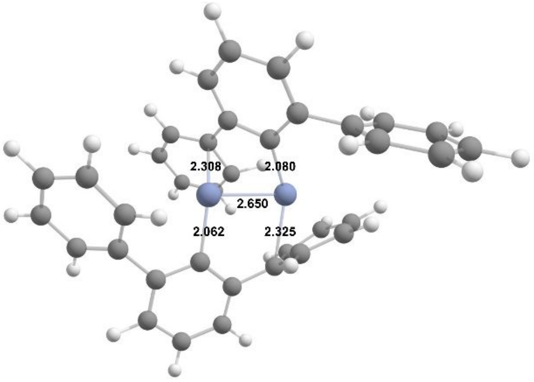

S = 3

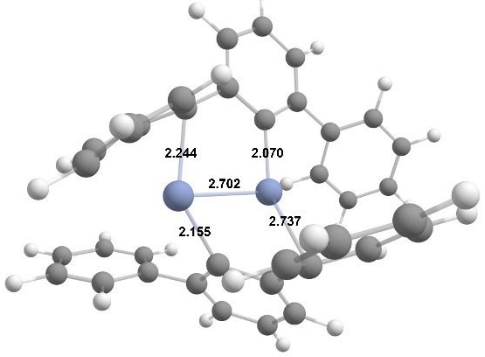

S = 4

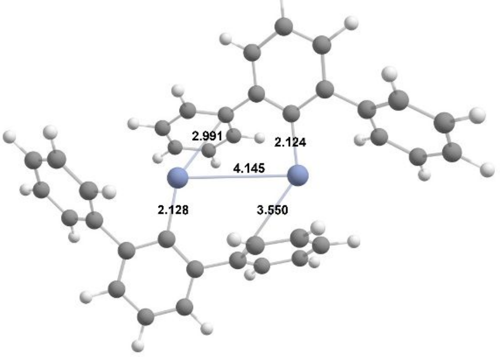

S = 5

Figure S6. Optimized geometries of **S** for different spin states by UB3LYP/def2-SVP method, figures made by Chemcraft[1]

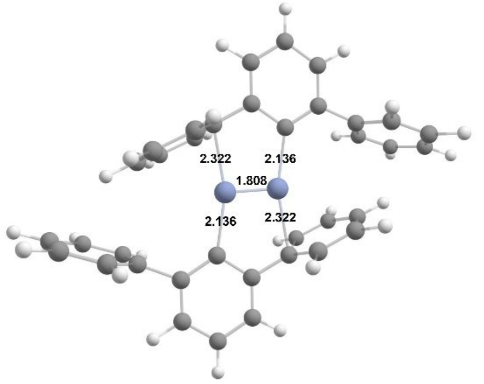

S “experimental”

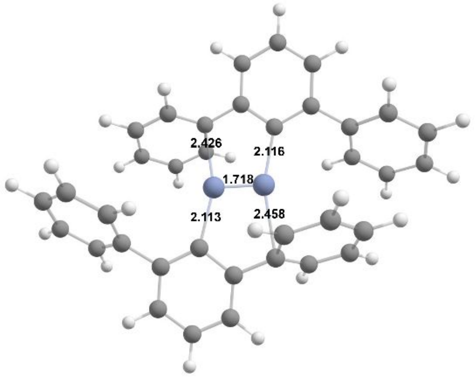

S = 0

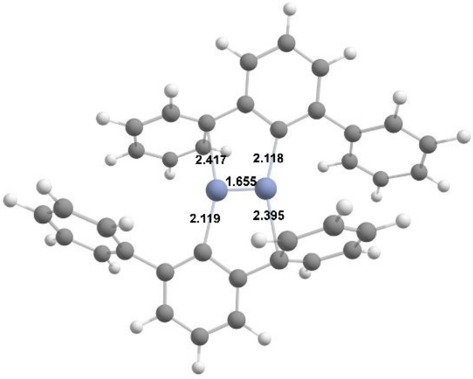

S = 0 (RB3LYP)

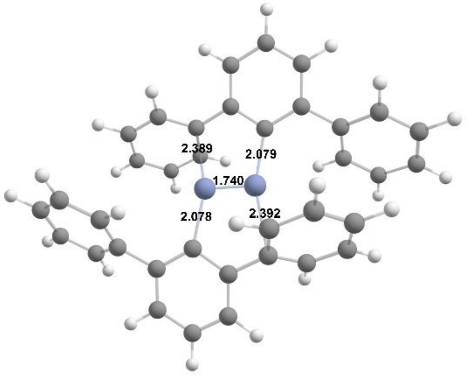

S = 1

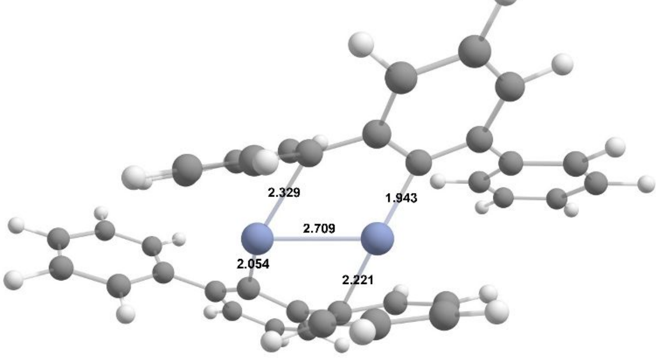

S = 2

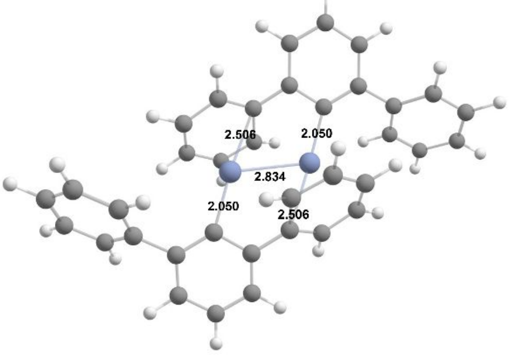

S = 3

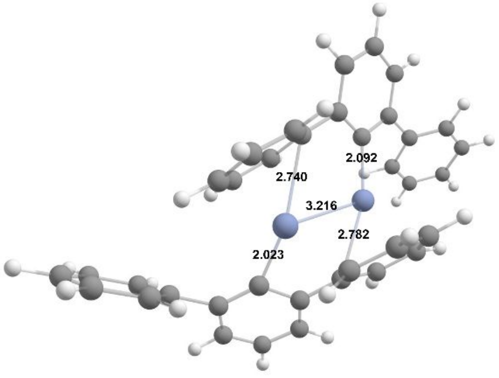

S = 4

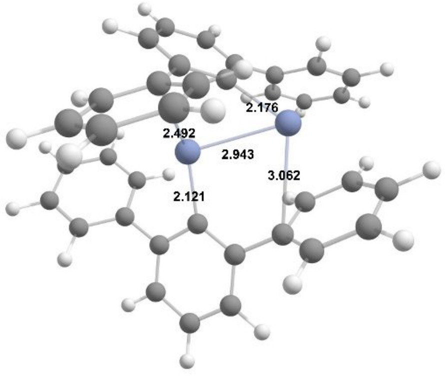

S = 5

Figure S7. Optimized geometries of **S** for different spin states by UBLYP/def2-SVP method, figures made by Chemcraft [1]

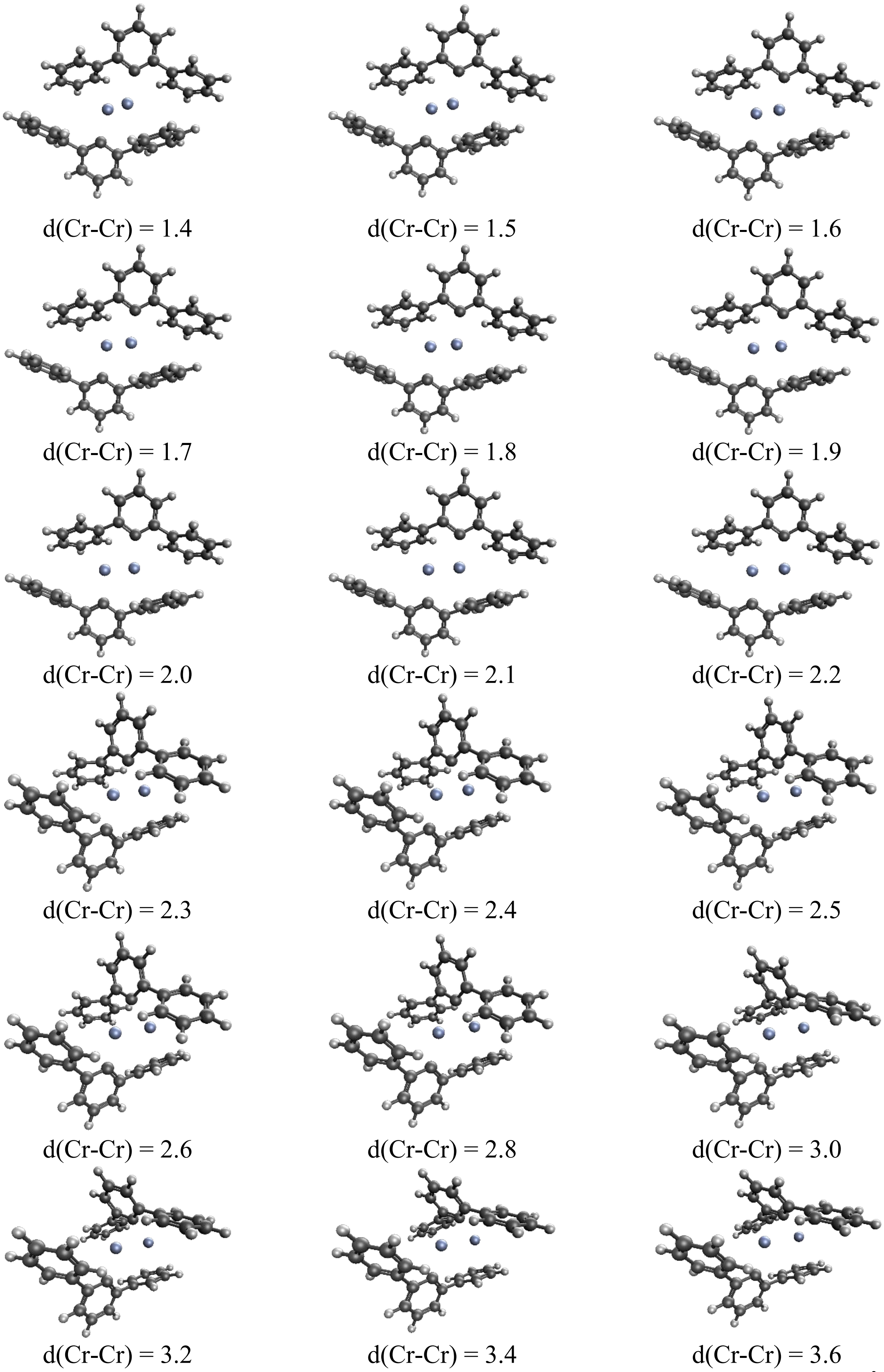

Figure S8. CAS(10, 10) optimized geometries of **S** for different frozen Cr-Cr distances [Å]

Table S27. Five main configurations of each spin state of CAS(10,10) S = 0 state-specific calculation of the optimized **S** complex at a Cr^{I}- Cr^{I} distance equal to 2.5 Å

Method	S	configuration	weight / %	$\Delta E_{CAS(10,10)}$/ kJ mol^{-1}
CAS(10,10)	0	$1\sigma^{2}1\pi^{1}1\pi^{*1}2\pi^{1}2\pi^{*1}1\delta^{1}1\delta^{*1}2\delta^{1}2\delta^{*1}$	2.8	0.00
		$1\sigma^{2}1\pi^{2}2\pi^{2}1\delta^{1}1\delta^{*1}2\delta^{1}2\delta^{1}$	2.2	
		$1\sigma^{2}1\pi^{2}2\pi^{2}1\delta^{2}2\delta^{2}$	1.9	
		$1\sigma^{1}1\sigma^{*1}1\pi^{2}2\pi^{1}2\pi^{*1}1\delta^{1}1\delta^{*1}2\delta^{1}2\delta^{*1}$	1.9	
		$1\sigma^{2}1\pi^{2}2\pi^{1}2\pi^{*1}1\delta^{2}2\delta^{1}2\delta^{*1}$	1.8	
CAS(10,10)	1	$1\sigma^{1}1\sigma^{*1}1\pi^{1}1\pi^{*1}2\pi^{1}2\pi^{*1}1\delta^{1}1\delta^{*1}2\delta^{1}2\delta^{*1}$	2.9	2.74
		$1\sigma^{2}1\pi^{2}2\pi^{1}2\pi^{*1}1\delta^{1}1\delta^{*1}2\delta^{1}2\delta^{*1}$	2.8	
		$1\sigma^{2}1\pi^{1}1\pi^{*1}2\pi^{2}1\delta^{1}1\delta^{*1}2\delta^{1}2\delta^{*1}$	2.6	
		$1\sigma^{2}1\pi^{1}1\pi^{*1}2\pi^{1}2\pi^{*1}1\delta^{2}2\delta^{1}2\delta^{*1}$	2.2	
		$1\sigma^{2}1\pi^{1}1\pi^{*1}2\pi^{1}2\pi^{*1}1\delta^{1}1\delta^{*1}2\delta^{2}$	2.1	
CAS(10,10)	2	$1\sigma^{2}1\pi^{1}1\pi^{*1}2\pi^{1}2\pi^{*1}1\delta^{1}1\delta^{*1}2\delta^{1}2\delta^{*1}$	4.9	8.26
		$1\sigma^{1}1\sigma^{*1}1\pi^{2}2\pi^{1}2\pi^{*1}1\delta^{1}1\delta^{*1}2\delta^{1}2\delta^{*1}$	3.2	
		$1\sigma^{1}1\sigma^{*1}1\pi^{1}1\pi^{*1}2\pi^{2}1\delta^{1}1\delta^{*1}2\delta^{1}2\delta^{*1}$	3.0	
		$1\sigma^{2}1\pi^{2}2\pi^{2}1\delta^{1}1\delta^{*1}2\delta^{1}2\delta^{*1}$	2.7	
		$1\sigma^{1}1\sigma^{*1}1\pi^{1}1\pi^{*1}2\pi^{1}2\pi^{*1}1\delta^{2}2\delta^{1}2\delta^{*1}$	2.6	
CAS(10,10)	3	$1\sigma^{1}1\sigma^{*1}1\pi^{1}1\pi^{*1}2\pi^{1}2\pi^{*1}1\delta^{1}1\delta^{*1}2\delta^{1}2\delta^{*1}$	8.5	16.66
		$1\sigma^{2}1\pi^{2}2\pi^{1}2\pi^{*1}1\delta^{1}1\delta^{*1}2\delta^{1}2\delta^{*1}$	5.6	
		$1\sigma^{2}1\pi^{1}1\pi^{*1}2\pi^{2}1\delta^{1}1\delta^{*1}2\delta^{1}2\delta^{*1}$	5.1	
		$1\sigma^{2}1\pi^{1}1\pi^{*1}2\pi^{1}2\pi^{*1}1\delta^{2}2\delta^{1}2\delta^{*1}$	4.3	
		$1\sigma^{2}1\pi^{1}1\pi^{*1}2\pi^{1}2\pi^{*1}1\delta^{1}1\delta^{*1}2\delta^{2}$	4.1	
CAS(10,10)	4	$1\sigma^{2}1\pi^{1}1\pi^{*1}2\pi^{1}2\pi^{*1}1\delta^{1}1\delta^{*1}2\delta^{1}2\delta^{*1}$	19.7	28.11
		$1\sigma^{1}1\sigma^{*1}1\pi^{2}2\pi^{1}2\pi^{*1}1\delta^{1}1\delta^{*1}2\delta^{1}2\delta^{*1}$	12.0	
		$1\sigma^{1}1\sigma^{*1}1\pi^{1}1\pi^{*1}2\pi^{2}1\delta^{1}1\delta^{*1}2\delta^{1}2\delta^{*1}$	10.9	
		$1\sigma^{1}1\sigma^{*1}1\pi^{1}1\pi^{*1}2\pi^{1}2\pi^{*1}1\delta^{2}2\delta^{1}2\delta^{*1}$	9.6	
		$1\sigma^{1}1\sigma^{*1}1\pi^{1}1\pi^{*1}2\pi^{1}2\pi^{*1}1\delta^{1}1\delta^{*1}2\delta^{2}$	9.3	
CAS(10,10)	5	$1\sigma^{1}1\sigma^{*1}1\pi^{1}1\pi^{*1}2\pi^{1}2\pi^{*1}1\delta^{1}1\delta^{*1}2\delta^{1}2\delta^{*1}$	100.0	42.91

Table S28. Composition of natural orbitals active space of CAS(10,10) S = 0 state-specific of **S** at [a]experimental geometry and optimized geometries of distances 1.7 Å and 2.5 Å between Cr(1)-Cr(2)

Orbital		140			141			142			143			144		
Cr(1)-Cr(2) / Å		1.80[a]	1.7	2.5	1.80[a]	1.7	2.5	1.80[a]	1.7	2.5	1.80[a]	1.7	2.5	1.80[a]	1.7	2.5
Cr(1)	s	0.0	0.0	5.8	0.0	0.0	0.0	2.0	0.4	1.4	4.1	5.0	1.4	0.0	0.0	0.0
	pz	0.2	0.1	-	1.5	1.1	-	0.0	0.0	-	0.0	0.0	-	-	-	0.0
	px	0.0	0.0	0.4	0.0	0.0	0.0	0.3	1.1	0.1	0.4	0.2	0.0	0.0	0.0	0.0
	py	0.8	0.8	0.1	0.1	0.1	0.0	0.0	0.1	0.0	0.0	0.0	0.0	0.0	0.0	0.0
	dz^2	0.0	0.3	0.2	0.0	2.3	24.7	3.2	9.3	0.0	42.3	31.0	22.6	0.0	3.4	1.1
	dxz	6.6	5.6	5.7	41.3	39.1	12.1	0.0	2.2	15.8	0.0	1.3	15.8	0.1	0.3	0.3
	dyz	0.0	0.4	5.4	0.0	1.0	1.5	0.0	0.1	2.7	0.0	3.7	0.0	47.5	43.1	39.1
	dx^2-y^2	0.0	1.4	12.9	0.0	1.8	11.7	43.6	34.4	15.7	1.6	8.2	8.5	0.0	0.7	0.1
	dxy	41.3	40.9	21.8	6.6	4.3	0.1	0.0	2.1	13.6	0.0	0.2	1.3	0.0	1.5	8.8
Cr(2)	s	0.0	0.0	6.0	0.0	0.0	0.0	2.0	0.4	1.4	4.1	5.0	1.7	0.0	0.0	0.0
	pz	0.2	0.1	0.1	1.5	1.1	0.2	0.0	0.0	0.0	0.0	0.0	0.0	-	-	0.0
	px	0.0	0.0	0.6	0.0	0.0	0.1	0.3	1.1	0.0	0.4	0.2	0.0	0.0	0.0	0.0
	py	0.8	0.8	-	0.1	0.1	-	0.0	0.1	-	0.0	0.0	-	0.0	0.0	0.0
	dz^2	0.0	0.3	0.7	0.0	2.3	23.2	3.2	9.3	2.2	42.3	30.9	20.0	0.0	3.4	0.4
	dxz	6.6	5.6	8.1	41.3	39.1	17.1	0.0	2.2	4.5	0.0	1.3	19.0	0.1	0.3	0.2
	dyz	0.0	0.4	0.8	0.0	1.0	1.0	0.0	0.1	8.6	0.0	3.7	1.4	47.5	43.1	37.0
	dx^2-y^2	0.0	1.4	25.9	0.0	1.8	7.6	43.6	34.4	4.9	1.6	8.2	3.3	0.0	0.7	0.0
	dxy	41.4	40.9	3.6	6.6	4.3	0.1	0.0	2.1	27.0	0.0	0.2	3.8	0.0	1.5	11.6

Orbital		145			146			147			148			149		
Cr(1)-Cr(2) / Å		1.80[a]	1.7	2.5	1.80[a]	1.7	2.5	1.80[a]	1.7	2.5	1.80[a]	1.7	2.5	1.80[a]	1.7	2.5
Cr(1)	s	0.0	0.0	0.0	0.6	0.5	0.5	0.2	0.1	0.3	0.0	0.0	0.0	0.1	0.2	0.7
	pz	-	-	0.0	-	-	0.0	-	-	0.0	-	-	0.2	-	-	0.4
	px	0.0	0.0	0.0	0.5	0.8	0.0	1.3	0.9	0.0	0.0	0.0	0.1	0.0	0.0	1.3
	py	0.0	0.0	0.0	0.1	0.2	0.0	0.1	0.1	0.2	0.0	0.0	0.0	0.1	0.0	0.5
	dz^2	0.0	3.3	1.1	46.2	34.9	23.0	2.5	8.2	0.0	0.0	2.3	24.3	0.0	0.2	0.4
	dxz	0.0	0.2	0.3	0.0	1.6	16.4	0.0	2.1	16.3	44.5	41.2	11.9	5.0	4.5	4.6
	dyz	48.7	43.9	39.2	0.0	3.9	0.0	0.0	0.1	2.7	0.0	1.2	1.5	0.0	0.5	5.7
	dx^2-y^2	0.0	0.8	0.1	1.6	7.5	8.3	44.3	35.7	15.6	0.0	1.8	11.6	0.0	1.6	12.5
	dxy	0.0	1.3	8.8	0.0	0.1	1.3	0.0	2.0	14.1	4.9	3.3	0.1	43.8	42.2	23.3
Cr(2)	s	0.0	0.0	0.0	0.6	0.5	0.9	0.2	0.1	0.8	0.0	0.0	0.0	0.1	0.2	3.7
	pz	-	-	0.0	-	-	0.0	-	-	0.0	-	-	0.0	-	-	0.1
	px	0.0	0.0	0.0	0.5	0.8	0.0	1.3	0.9	0.0	0.0	0.0	0.0	0.0	0.0	0.4
	py	0.0	0.0	0.0	0.1	0.2	0.1	0.1	0.1	0.1	0.0	0.0	0.0	0.1	0.0	0.1
	dz^2	0.0	3.3	0.4	46.2	34.9	20.7	2.5	8.2	2.4	0.0	2.3	23.7	0.0	0.2	1.2
	dxz	0.0	0.2	0.2	0.0	1.6	19.6	0.0	2.1	4.7	44.5	41.2	17.5	5.0	4.5	8.0
	dyz	48.7	43.9	37.2	0.0	3.9	1.3	0.0	0.1	8.7	0.0	1.2	1.0	0.0	0.5	1.1
	dx^2-y^2	0.0	0.8	0.0	1.6	7.5	3.5	44.3	35.7	4.9	0.0	1.8	7.8	0.0	1.6	29.4
	dxy	0.0	1.3	11.7	0.0	0.1	3.6	0.0	2.0	28.0	4.9	3.3	0.1	43.8	42.2	4.8

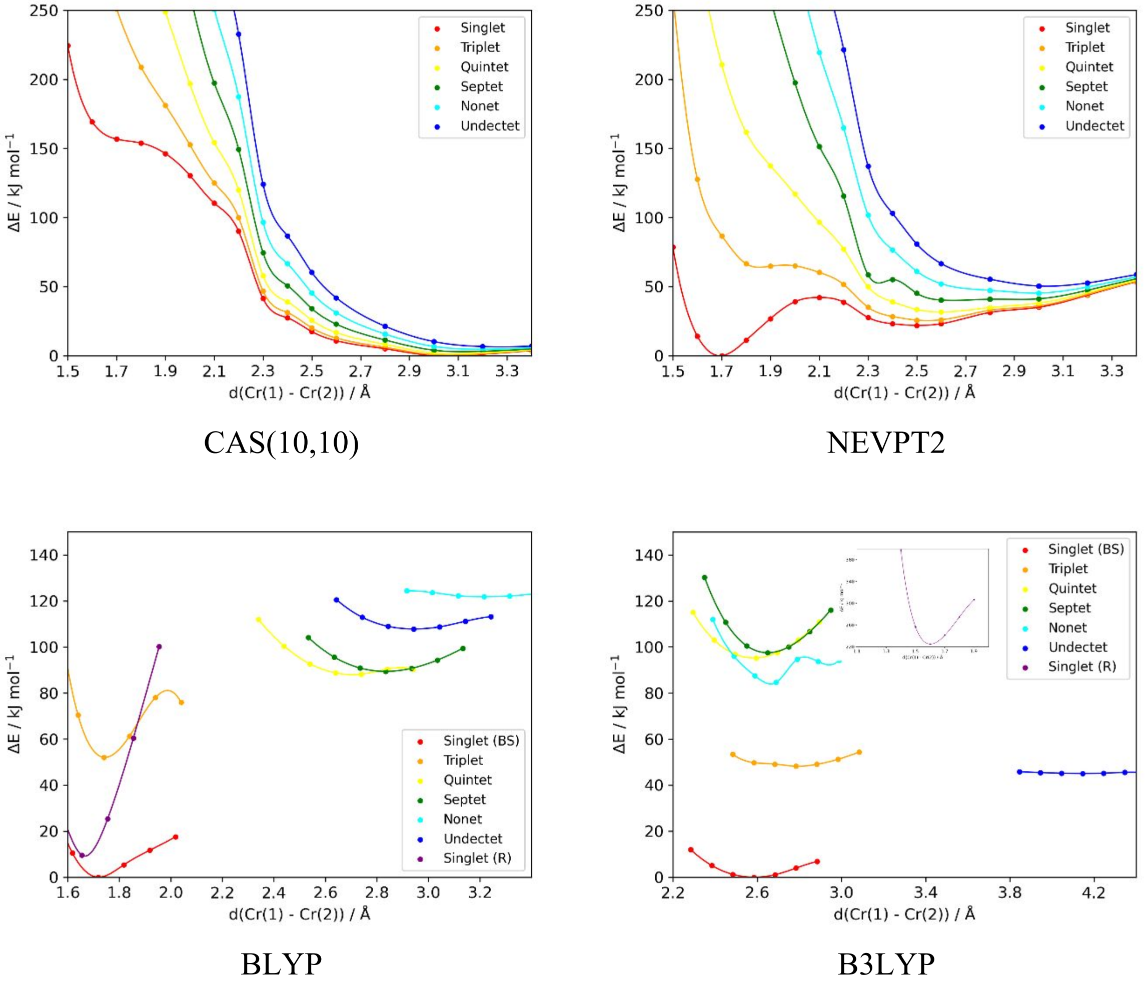

Figure S9. CAS(10,10) and NEVPT2 state-specific S = 0 potential energy curves and BLYP and B3LYP scans around respective equilibria of **S**, BS = broken-symmetry, R = restricted

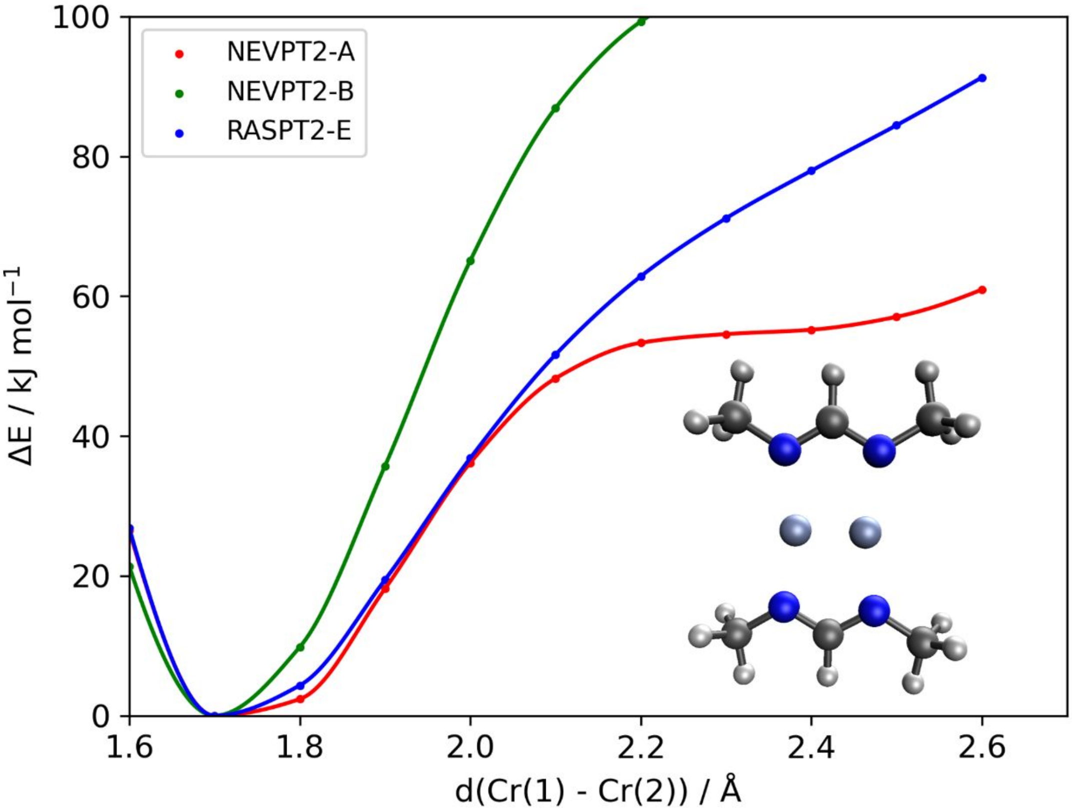

Figure S10. Comparison of PEC of the diamidinate Cr^{I}-Cr^{I} system of Huang *et al*. [2], NEVPT2 represents calculation at (10,10) active space and def2-SVP basis set (denoted as NEVPT2-A) and def2-TZVPP basis set (denoted as NEVPT2-B), RASPT2-E is the diamidinate Cr^{I}-Cr^{I} calculation with an (12,22) active space and the ANO-RCC basis set (including two h functions for Cr atoms) of Huang *et al*. [2]

References

[1] Chemcraft - graphical software for visualization of quantum chemistry computations. Version 1.8, build 682. https://www.chemcraftprog.com

[2] G.T. Huang, J.S.K. Yu, Analyses on Molecular Properties of the Diamidinate Cr^{I}-Cr^{I} Complex by Multireference and DFT Approaches, Journal of Physical Chemistry A 123 (2019) 7803–7813. https://doi.org/10.1021/acs.jpca.9b04423.
